# Innovative Design of PEI‐Modified AMO‐Layered Double Hydroxide for Efficient and Stable Direct Air Capture of CO_2_

**DOI:** 10.1002/advs.202507756

**Published:** 2025-06-30

**Authors:** Meng Zhao, Liang Huang, Yanshan Gao, Ziling Wang, Xuancan Zhu, Qiang Wang, Dermot O'Hare

**Affiliations:** ^1^ College of Environmental Science and Engineering Beijing Forestry University Beijing 100083 China; ^2^ State Key Laboratory of Efficient Production of Forest Resources Beijing Forestry University Beijing 100083 China; ^3^ Research Center of Solar Power & Refrigeration Institute of Refrigeration and Cryogenics Shanghai Jiao Tong University Shanghai 200240 China; ^4^ Chemistry Research Laboratory Department of Chemistry University of Oxford Mansfield Road Oxford OX1 3TA UK

**Keywords:** aqueous miscible organic solvent treatment, direct air capture, layered double hydroxide, solid amine adsorbents, surface hydroxyl groups

## Abstract

Emerging as a critical technology for atmospheric carbon dioxide (CO_2_) removal, the mass deployment of direct air capture (DAC) demands breakthrough innovations in efficient and stable adsorbent materials that simultaneously achieve high capacity, oxidative durability, and low cost. Herein, a hydroxyl‐rich Mg_0.55_Al layered double hydroxide (LDH) support is developed via aqueous miscible organic solvent treatment, circumventing energy‐intensive calcination while engineering mesopores for efficient polyethyleneimine (PEI) loading. The optimized 60 wt.% PEI modified Mg_0.55_Al‐CO_3_ AMO‐LDH achieves a CO_2_ uptake of 3.92 mmol g^−1^ under simulated wet air at 25 °C and retains 90.8% capacity over 20 cycles. Crucially, the abundant surface hydroxyls of uncalcined LDH, validated by ^1^H Nuclear Magnetic Resonance and in situ X‐ray Photoelectron Spectroscopy, form hydrogen bonds with PEI, suppressing oxidative degradation. After 3 h at 120 °C in simulated air, PEI‐LDH retains a CO_2_ capacity of 1.06 mmol g^−1^, significantly outperforming PEI/mixed metal oxide and conventional silica‐based adsorbents. In situ Diffuse Reflectance Infrared Fourier Transform Spectroscopy further reveals that hydroxyl‐mediated amine anchoring minimizes water co‐adsorption. This work establishes a dual strategy of hydroxyl preservation and mesopore engineering to design cost‐effective DAC adsorbents, achieving both high capacity and exceptional stability under realistic operating conditions.

## Graphical abstract

An uncalcined, hydroxyl‐rich MgAl layered double hydroxide, synthesized via low‐energy aqueous miscible organic solvent treatment, enables mesopore‐engineered polyethyleneimine anchoring for direct air capture (DAC). The exfoliated structure exposes abundant hydroxyls that stabilize amines via hydrogen bonding, effectively suppressing oxidative degradation and humidity sensitivity. The sorbent captures 3.92 mmol g^−1^ carbon dioxide under humid air and retains >90% capacity over 20 cycles, addressing energy and stability barriers in DAC.

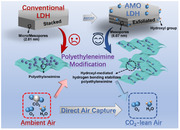

## 1 Introduction

Carbon dioxide (CO_2_) as the most abundant and persistent greenhouse gas (GHG), significantly contributes to global warming and disrupts the climate system.^[^ ^1^ ^]^ In response to this critical issue, carbon capture technologies have garnered great attention.^[^ ^2^ ^]^ Among these, Direct Air Capture (DAC) has emerged as a promising approach to reduce atmospheric CO_2_ concentrations.^[^ ^3^ ^]^ Unlike conventional capture methods that focus on point‐source emissions, DAC targets CO_2_ directly from ambient air, offering the potential to remove excess CO_2_ from the atmosphere and provide a long‐term solution to climate change.^[^ ^4^ ^]^ Nowadays, efficient CO_2_ adsorbents is highly demanded to make DAC technology economically viable and scalable for widespread deployment. Although liquid amine absorption has been validated at large scales, it still faces significant challenges such as high energy consumption, absorbent degradation, and secondary pollution.^[^ ^5^ ^]^ Therefore, there is an increasing demand for more efficient and sustainable carbon capture solutions.

To address these challenges, solid amine adsorbents have been developed by immobilizing liquid amines onto porous supporting materials.^[^ ^6^ ^]^ Compared to liquid‐phase absorption, solid amine adsorption offers lower heat capacity, smaller regeneration temperature/pressure swings, and reduced secondary pollution. These improvements can lower operational costs and regeneration energy consumption. However, designing solid amine materials for DAC remains a complex task, requiring a balance between capacity, kinetics, selectivity, long‐term stability, and scalability. These properties of solid amine‐based materials are strongly influenced by the porosity and surface hydroxyl groups of supports. Commonly used porous supports for solid amine adsorbents include Al_2_O_3_,^[^ ^7^ ^]^ silica,^[^ ^3^, ^8^ ^]^ metal–organic frameworks (MOFs),^[^ ^9^ ^]^ and covalent organic frameworks (COFs).^[^ ^10^ ^]^

Recently, mixed metal oxide (MMO) has been demonstrated as an effective supporting substrate for solid amine adsorbents for DAC. Zhu et al.^[^ ^11^ ^]^ synthesized a novel class of DAC adsorbents by loading 67 wt.% branched polyethylenimine (PEI) onto Mg/Al‐O MMOs. These materials exhibited unexpectedly high CO_2_ uptake (2.27 mmol g^−1^), rapid kinetics (1.1 mmol g^−1^ h^−1^), and remarkable stability over 20 cycles at 25 °C under 0.4 mbar CO_2_. Later, we explored the use of tetraethylenepentamine (TEPA) as an alternative to PEI, and the obtained Mg_0.55_Al‐O‐TEPA67% adsorbent showed even higher CO_2_ uptake (3.0 mmol g^−1^) and excellent regenerability under the same condition, maintaining 90% of the initial adsorption capacity after 80 adsorption/desorption cycles.^[^ ^12^ ^]^ The amine‐impregnated MMOs exhibited enhanced CO_2_ capacity and stability in the presence of moisture, making them suitable for steam‐assisted regeneration. Furthermore, the cost‐effectiveness and scalability of MMOs enhance their potential for large‐scale DAC applications.

While MMO‐supported amines show potential for CO_2_ capture, recent comprehensive reviews have emphasized the need for adsorbents that combine tailored porosity, low regeneration energy, and long‐term operational stability across a wide range of environmental conditions—features essential for scalable DAC deployment.^[^ ^13^ ^]^ In particular, amine‐functionalized materials with abundant surface hydroxyl groups or chemical functionalities have emerged as strong candidates to overcome challenges such as moisture sensitivity and oxidative degradation, which remain key bottlenecks in real‐world DAC operations.^[^ ^14^ ^]^

Our recent work^[^ ^15^ ^]^ reveals that surface hydroxyl groups—abundant in uncalcined layered double hydroxides (LDHs)—critically enhance amine stability by forming protective hydrogen bonds. This finding motivated us to bypass energy‐intensive calcination (which converts LDH to MMO) and directly exploit LDH's native hydroxyl‐rich surfaces for amine immobilization.^[^ ^16^ ^]^ However, conventional LDHs suffer from limited porosity and surface area, which severely restricts their potential for amine immobilization and dispersion.

In this work, we employed an aqueous miscible organic solvent treatment (AMOST) strategy to engineer LDH mesoporosity (average pore radius: 8.07 nm) while preserving surface hydroxyl groups.^[^ ^17^ ^]^ We proved that the obtained PEI/AMOST‐LDH adsorbent can achieve dual optimizations: 1) a tailored pore architecture that accommodates 60 wt.% PEI loading without pore blockage, enabling a CO_2_ uptake of 3.92 mmol·g^−1^ under simulated DAC conditions (400 ppm CO_2_, 21% O_2_, 3% H_2_O), while maintaining 90.8% of its initial capacity after 20 adsorption‐desorption cycles; 2) enhanced amine‐support interactions via hydroxyl‐mediated hydrogen bonding, which significantly suppresses H_2_O co‐adsorption and improves oxidative stability, outperforming both MMO‐supported and silica‐based benchmarks. By integrating tailored mesoporosity with hydroxyl‐mediated amine stabilization, this study provides a new direction for developing high‐performance and low‐cost DAC adsorbents, achieving synergistic optimization of CO_2_ uptake and long‐term durability while demonstrating superior performance under practical DAC conditions.

## 2 Results and Discussion

### 2.1 Structural and Textural Features of AMOST‐LDHs

**Figure 1:**
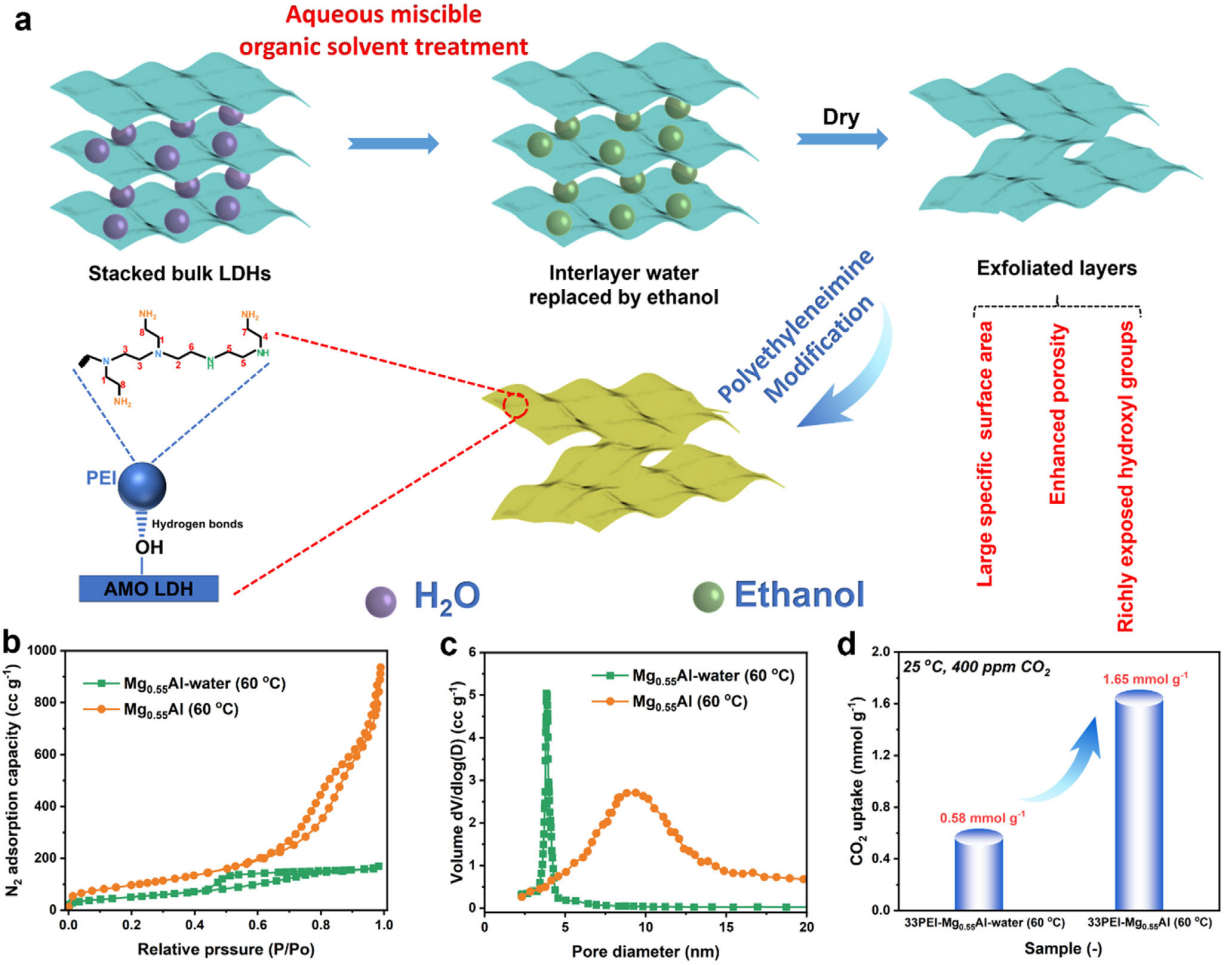
a) Schematic illustration of the aqueous miscible organic solvent treatment (AMOST) process for synthesizing Mg_0.55_Al layered double hydroxide (LDH) and subsequent polyethyleneimine (PEI) impregnation. b) N_2_ adsorption–desorption isotherms and c) pore size distributions of Mg_0.55_Al‐CO_3_ LDH synthesized via conventional coprecipitation (denoted Mg_0.55_Al‐water (60 °C)) versus the AMOST method (Mg_0.55_Al (60 °C)). d) CO_2_ uptakes at 25 °C under 400 ppm CO_2_ for 33 wt.% PEI‐impregnated composites: 33PEI‐Mg_0.55_Al‐water (60 °C) and 33PEI‐Mg_0.55_Al (60 °C).

AMOST treatment of water wet LDH offers a transformative strategy for optimizing the textural characteristics of Mg_0.55_Al‐CO_3_ LDH, markedly enhancing its porosity and specific surface area.^[^ ^18^ ^]^ These structural modifications critically govern the dispersion efficiency of PEI and its resultant CO_2_ adsorption performance. As illustrated in **Figure** **1**a, Mg_0.55_Al‐water (60 °C), a LDH sample synthesized via the conventional coprecipitation method, washed with deionized water and vacuum‐dried at 60 °C without calcination, exhibits a densely packed lamellar morphology with limited porosity and minimal accessible surface area, which hinders effective PEI immobilization. In contrast, Mg_0.55_Al (60 °C), synthesized via the AMOST method, undergoes ethanol rinsing and redispersion before filtration, disrupting interlayer hydrogen bonding by replacing interlayer and surface‐adsorbed water molecules with ethanol.^[^ ^18^ ^]^ Subsequent drying induces rapid ethanol evaporation from interlayers, triggering structural expansion and exfoliation. Notably, the markedly lower surface tension of ethanol compared to water results in significantly reduced capillary pressure during drying. This minimized capillary stress helps preserve mesoporosity and inhibits restacking of exfoliated nanosheets, thereby contributing to the formation of a stable, open‐layered architecture.^[^ ^19^ ^]^ The AMOST process produces a highly exfoliated and dispersed nanoplatelet architecture with abundant mesopores, which are essential for PEI anchoring and CO_2_ chemisorption.^[^ ^20^ ^]^

N_2_ adsorption/desorption analysis corroborates the substantial textural enhancement, with AMOST LDH exhibiting a 3.6‐fold increase in pore volume (1.64 vs 0.36 cc g^−1^ for a conventional LDH) and a 1.9‐fold increase in specific surface area (358.43 vs 188.49 m^2^ g^−1^). Critically, the mesopore‐dominated architecture demonstrates a tripling of the average pore radius from 2.81 nm (conventional LDH) to 8.07 nm (AMOST LDH), attaining pore structural characteristics comparable to thermally derived MMO while eliminating the need for energy‐intensive calcination (Figure 1b,c; Table S1, Supporting Information). A comparative summary in Table S2 (Supporting Information) further highlights the textural advantages of the AMOST strategy over conventional LDH synthesis routes.

This engineered structural transition enhances CO_2_ diffusion kinetics by reducing mass transfer resistance and provides spatially uniform domains for PEI immobilization. After 33 wt.% PEI loading, AMOST‐LDH retains substantial porosity (157.75 m^2^ g^−1^ surface area, 0.90 cm^3^g^−1^ pore volume), whereas a conventional LDH, with the same PEI loading, exhibits negligible surface area and pore volume with viscous agglomeration, indicating severe PEI accumulation due to pore blockage (Figure S1 and Table S3, Supporting Information). This pore topology optimization enables the 33PEI‐Mg_0.55_Al (60 °C) composite to achieve a CO_2_ uptake of 1.65 mmol g^−1^ under simulated air conditions (25 °C, 400 ppm CO_2_), outperforming its water‐washed counterpart by 300% (Figure 1d). The synergistic interplay between enlarged mesoporous channels and amplified surface accessibility facilitates both amine dispersion homogeneity and gas‐amine interaction efficiency, underscoring the pivotal role of pore geometry engineering in adsorbent performance.

The AMOST‐induced structural engineering establishes a paradigm for tailoring LDH‐amine interfaces, simultaneously boosting adsorption capacity by mitigating pore blockage. Moreover, AMOST increases the exposure of surface hydroxyl groups, further reinforcing amine anchoring. These advances position AMOST LDHs as a sustainable, high‐performance alternative to conventional calcined MMOs for DAC. Subsequent analyses will systematically compare hydroxyl‐mediated PEI anchoring mechanisms in AMOST‐LDH versus thermally derived MMOs.

### 2.2 Characterization and CO_2_ Adsorption Performance of PEI‐Mg_0.55_Al LDH for Direct Air Capture

**Figure 2:**
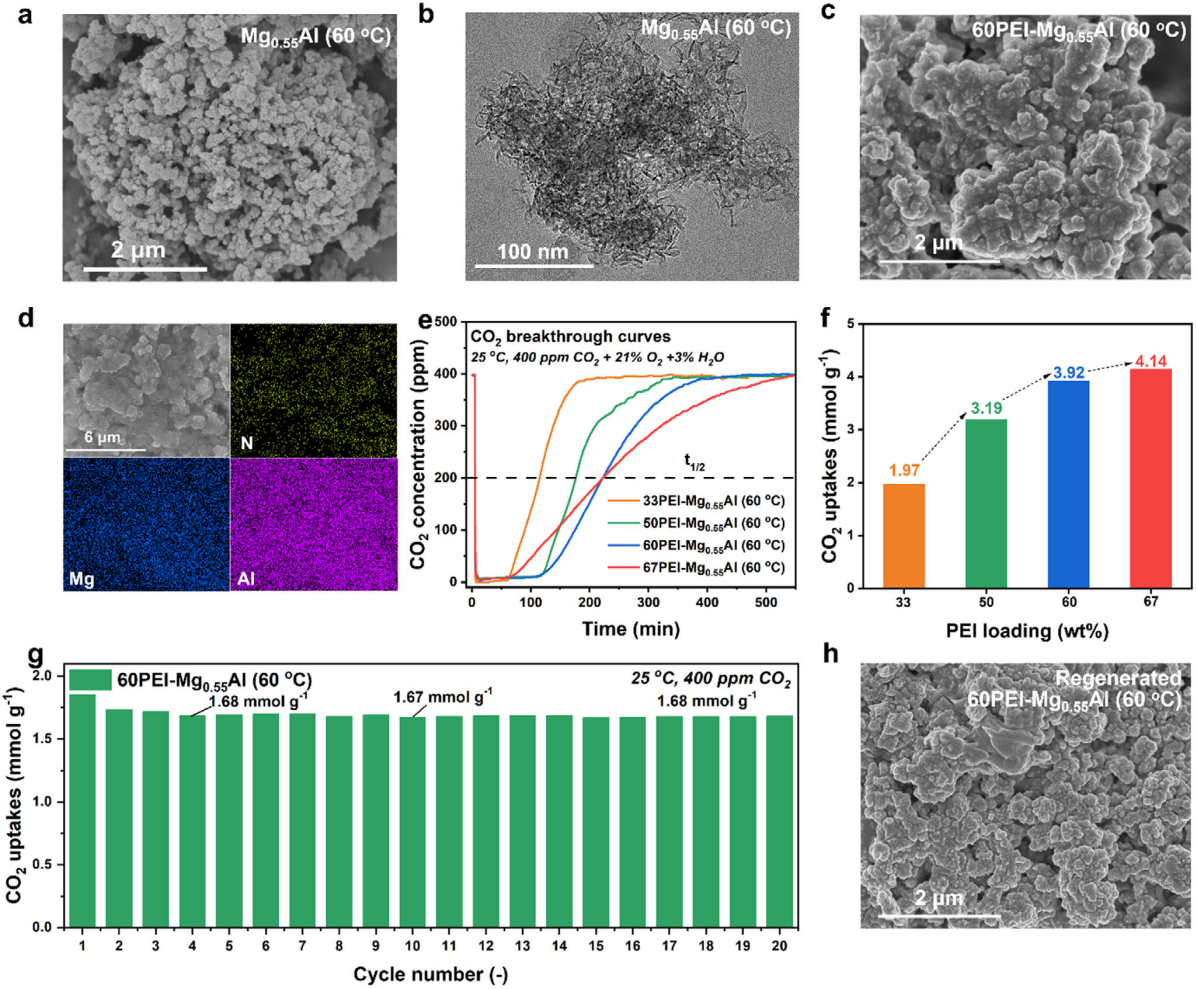
a) Scanning electron microscopy (SEM) images of Mg_0.55_Al (60 °C); b) Transmission electron microscopy images of Mg_0.55_Al (60 °C); c) SEM images of 60PEI‐Mg_0.55_Al (60 °C); d) energy dispersive X‐ray spectroscopy results of 60PEI‐Mg_0.55_Al (60 °C); e) breakthrough curves for *x*PEI‐Mg_0.55_Al (60 °C) during CO_2_ adsorption at 25 °C in a gas mixture of 400 ppm CO_2_, 21% O_2_, and 3% H_2_O; f) CO_2_ adsorption capacity of the xPEI‐Mg_0.55_Al (60 °C), calculated from the breakthrough curves; g) CO_2_ adsorption and desorption cycles for 60PEI‐Mg_0.55_Al (60 °C) at 25 °C under 400 ppm CO_2_ for 180 min, followed by desorption at 120 °C for 15 min under N_2_; h) SEM image of the regenerated 60PEI‐Mg_0.55_Al (60 °C) sample after 20 cycles.

Building upon the synthesis methodology outlined in Section 2.1, the structural and functional properties of the Mg_0.55_Al (60 °C) LDH were systematically investigated. The AMOST method, combined with vacuum drying, effectively eliminated interlayer water, weakening interlayer interactions and promoting the exfoliation of LDH into ultrathin nanosheets. Scanning electron microscopy (SEM) and transmission electron microscopy (TEM) images confirmed the formation of highly dispersive nanosheets with a flower‐like morphology and slit‐shaped mesopores (**Figure** **2**a,b).^[^ ^21^ ^]^ This structural evolution significantly increased the accessibility of surface Mg/Al hydroxyl groups, which served as anchoring sites for the homogeneous dispersion of PEI across the LDH matrix.

Following 60 wt.% PEI loading, the SEM image of the composite revealed a denser morphology with partial aggregation, suggesting effective pore filling and surface adherence of PEI (Figure 2c). To substantiate this qualitative observation, N_2_ adsorption–desorption measurements were conducted. As summarized in Table S3 and Figure S1 (Supporting Information), the total pore volume progressively decreased to 0.20, 0.07, and 0.03 cc g^−1^ for 50, 60, and 67 wt.% PEI‐loaded samples, respectively, while the specific surface area dropped to 30.19, 11.06, and 6.02 m^2^ g^−1^. Correspondingly, the nitrogen adsorption isotherms evolved from a characteristic Type IV shape to a nearly flat profile at 67 wt.% (Figure S1a, Supporting Information), and the pore size distributions became increasingly featureless (Figure S1b, Supporting Information), indicating substantial pore blockage, particularly at 67 wt.% PEI loading. The average pore radius showed a non‐monotonic trend—increasing initially due to preferential filling of small pores and then decreasing to 10.79 and 8.91 nm at 60 and 67 wt.%, respectively—as pore occlusion became more pronounced.^[^ ^12^ ^]^ These textural evolutions collectively validate the SEM‐inferred pore‐filling behavior and confirm progressive intrusion of PEI into the pore channels of the LDH framework.

Despite this aggregation, the flower‐like architecture remained intact, preserving the hierarchical porosity critical for gas diffusion. Energy‐dispersive X‐ray spectroscopy (EDS) elemental mapping further validated the uniform spatial distribution of nitrogen (N), magnesium (Mg), and aluminum (Al), confirming successful PEI immobilization without compromising the structural integrity of the Mg_0.55_Al framework (Figure 2d). Such uniformity, together with the preserved hierarchical porosity (average pore radius of 10.79 nm and residual surface area of 11.06 m^2^ g^−1^ at 60 wt.% PEI), ensures sufficient exposure and accessibility of amine sites, which is essential for efficient CO_2_ capture under ultra‐dilute atmospheric conditions.

The CO_2_ adsorption capacity of the *x*PEI‐Mg_0.55_Al LDH series for DAC was assessed through breakthrough experiments conducted under humidified air conditions (400 ppm CO_2_, 21% O_2_, and 3% H_2_O). As shown in Figure 2e,f, CO_2_ uptake increased with PEI loading, reaching 3.92 and 4.14 mmol g^−1^ for 60  and 67 wt.% PEI loadings, respectively. These values are among the highest reported for amine‐impregnated DAC adsorbents (**Table** **1**), as further supported by the comparative summary in Table S4 (Supporting Information), which benchmarks the present materials against recently reported representative high‐performance amine‐functionalized LDHs at 25 °C. However, the 67 wt.% PEI composite exhibited premature breakthrough due to pore occlusion, which restricted CO_2_ diffusion kinetics.

**Table 1:** CO_2_ Uptakes of Amine‐impregnated Adsorbents for Direct Air Capture.

Support	Amine	Loadings	Adsorption conditions	Method	Capacity [mmol g^−1^]	Refs.
COF	PEI (Mw 600)	9.24 mmol N g^−1^	Dry, 25 °C, 400 ppm CO_2_/N_2_	CO_2_ breakthrough test	0.48	[10]
COF	PEI (Mw 600)	9.24 mmol N g^−1^	75% RH, 25 °C, 400 ppm CO_2_/N_2_	CO_2_ breakthrough test	1.24	[10]
Resin HP2MGL	PEI (Mw 600)	50 wt.%	10% RH, 25 °C, 400 ppm CO_2_/20.5% O_2_/CO_2_/79.5% N_2_	CO_2_ breakthrough test	2.44	[22]
SBA‐15	PEI (Mw 800)	50 wt.%	Dry, 30 °C, 400 ppm CO_2_/He	TGA	1.55	[23]
γ‐Alumina	PEI (Mw 800)	48 wt.%	Dry, 25 °C, 400 ppm CO_2_/Ar	TGA	1.74	[24]
MCF	PEI (Mw 1200)	63 wt.%	Dry, 46 °C,420 ppm CO_2_/N_2_	CO_2_ breakthrough test	1.94	[25]
MCF	PEI (Mw 1200)	63 wt.%	20%RH, 46 °C, 420 ppm CO_2_/N_2_	CO_2_ breakthrough test	2.52	[25]
PE‐MCM‐41	PEI (Mw 800)	40 wt.%	Dry, 25 °C, 400 ppm CO_2_/N_2_	CO_2_ breakthrough test	2.18	[26]
PE‐MCM‐41	PEI (Mw 800)	40 wt.%	64% RH, 25 °C, 400 ppm CO_2_/N_2_	CO_2_ breakthrough test	2.92	[26]
MIL‐101(Cr)	PEI (Mw 800)	60 wt.%	Dry, 25 °C 400 ppm CO_2_/He	TGA	1.04	[27]
MIL‐100 (Cr)		PEI (Mw 600)	50 wt.%	Dry, 25 °C 400 ppm CO_2_/N_2_	TGA	1.21	[9c]
MMO	PEI (Mw 600)	67 wt.%	3% H_2_O, 25 °C, 400 ppm CO_2_/N_2_	CO_2_ breakthrough test	2.27	[11]
MMO	TEPA	67 wt.%	Dry, 25 °C, 400 ppm CO_2_/N_2_	TGA	3.0	[12]
LDH	PEI (Mw 600)	60 wt.%	3% H_2_O, 25 °C, 400 ppm CO_2_/21% O_2_/N_2_	CO_2_ breakthrough test	3.92	This work
LDH	PEI (Mw 600)	67 wt.%	3% H_2_O, 25 °C, 400 ppm CO_2_/21% O_2_/N_2_	CO_2_ breakthrough test	4.14	This work

^a)^ MCF: mesocellular foam;

^b)^ TGA: thermogravimetric analysis;

^c)^ PE‐MCM‐41: pore‐expanded MCM‐41.

aTo gain further insight into the adsorption kinetics, we analyzed the CO_2_ breakthrough curves and extracted the half‐breakthrough time (t_1/2_) as a representative kinetic parameter (Figure 2e). The t_1/2_ increased from 109 min for 33PEI to 171 min for 50PEI, reaching a maximum of 216 min for 60PEI. Notably, increasing PEI content to 67 wt.% did not further enhance kinetics, with t_1/2_ remaining nearly constant at 216 min, indicating that excessive PEI loading causes diffusion limitations due to pore blockage. Time‐resolved CO_2_ uptake calculated from breakthrough curves (Figure S2, Supporting Information) also revealed slower adsorption rates for the 67 wt.% PEI sample beyond 180 min, further supporting the presence of mass transfer resistance despite its higher theoretical capacity.

Overall, these results demonstrate that the adsorption kinetics are governed by a balance between CO_2_ affinity (enhanced by increasing amine content) and diffusion efficiency (limited by excessive pore filling). The 60 wt.% PEI‐Mg_0.55_Al (60 °C) adsorbent demonstrated an optimal balance between amine site density, pore accessibility, and adsorption kinetics, achieving a capacity of 3.92 mmol g^−1^ with sustained structural stability. This performance highlights its potential as a scalable material for energy‐efficient DAC systems.

### 2.3 Cycling Stability and Reproducibility of 60PEI‐Mg_0.55_Al (60 °C) Adsorbent

Following the optimization of the 60PEI‐Mg_0.55_Al (60 °C) adsorbent in Section 2.2, its long‐term cycling stability, a critical metric for any commercializable DAC technology, was evaluated under simulated adsorption‐desorption conditions. Cyclic thermogravimetric analysis (TGA) was conducted using a protocol comprising three stages: 1) pretreatment (120 °C, N_2_ flow, 10 mg sample), 2) adsorption (25 °C, 400 ppm CO_2_/N_2_ flow, 180 min), and 3) desorption (120 °C, N_2_ flow, 15 min), repeated over 20 cycles (Figure 2g).

The adsorbent demonstrated exceptional durability, retaining 90.82% of its initial CO_2_ uptake capacity (1.85 mmol g^−1^) after 20 cycles. A pronounced yet predictable performance decline of 6.39% occurred during the first two cycles (1.85 to 1.73 mmol g^−1^), attributed to irreversible interlayer carbonate (CO_3_^2−^) formation within the Mg_0.55_Al LDH framework. This phenomenon arises from the strong affinity of the positively charged LDH layers for CO_2_‐derived carbonate species, which remain thermodynamically stable below 300 °C. Subsequent cycles exhibited minimal capacity loss (2.98% over 19 cycles), stabilizing at 1.68 mmol g^−1^ by the 20th cycle, confirming the robustness of the PEI‐LDH interface under mild regeneration conditions.

Post‐cycling characterization revealed no structural degradation. SEM imaging confirmed retention of the flower‐like morphology without particle agglomeration (Figure 2h), while EDS mapping verified homogeneous N, Mg, and Al distributions (Figure S3, Supporting Information), corroborating the stability of PEI dispersion and LDH integrity. These findings underscore the synergistic interplay between the Mg_0.55_Al LDH host and PEI guest, where the LDH's hydroxyl‐rich surface mitigates amine leaching while its layered structure accommodates reversible CO_2_ capture. The combination of high cyclic stability (>90% retention), fast regeneration kinetics (15 min), and structural resilience positions the 60PEI‐Mg_0.55_Al (60 °C) nanocomposite as a technologically viable adsorbent for energy‐efficient DAC systems.

In addition to cyclic stability, the reproducibility and batch‐to‐batch consistency of the 60PEI‐Mg_0.55_Al (60 °C) adsorbent were assessed by thermogravimetric CO_2_ adsorption tests conducted at 25 °C under 400 ppm CO_2_. Repeated measurements on the same synthesis batch yielded CO_2_ uptake values with a relative standard deviation (RSD) of 2.66%, while samples from four independently synthesized batches showed an RSD of 2.41% (Table S5, Supporting Information). These results confirm excellent measurement precision and synthesis reliability, which are essential for scalable production and practical DAC deployment.

### 2.4 Oxidation Resistance of PEI‐Modified Mg_0.55_Al‐CO_3_ AMO‐LDH

The oxidative stability of PEI‐functionalized adsorbents is pivotal for their practical application in DAC, where prolonged oxygen exposure during cyclic operations demands robust structural and chemical resilience.^[^ ^28^ ^]^ To evaluate this property in Mg_0.55_Al LDH‐supported PEI composites, oxidative aging tests were systematically performed on samples synthesized with LDH calcination temperatures ranging from 60 to 400 °C.

The Fourier transform infrared (FTIR) spectra of 60 wt.% PEI‐modified Mg_0.55_Al (*y*) adsorbents revealed distinct vibrational signatures indicative of PEI immobilization and interfacial interactions (Figure S4, Supporting Information). Characteristic peaks at 2931 and 2811 cm^−1^ were attributed to the asymmetric and symmetric C─H stretching vibrations of PEI's alkyl chains, while bands at 3350 and 3282 cm^−1^ were assigned to the N─H stretching modes of primary (─NH_2_) and secondary (─NH─) amines. Additional features at 1579 and 1454 cm^−1^ were ascribed to N─H bending vibrations coupled with C─H bending. Peaks at 1303 and 1122 cm^−1^ were tentatively linked to carbamate species (NCOO⁻ skeletal vibrations) and C─O─C stretching, likely originating from pre‐adsorbed CO_2_ during ambient exposure.^[^ ^7^ ^]^ Notably, a broad absorption band spanning 3000–3700 cm^−1^ in the 60PEI‐Mg_0.55_Al (60 °C) indicated overlapping O─H (LDH surface hydroxyls) and N─H stretching vibrations. The pronounced broadening and enhanced intensity of this band suggest robust hydrogen bonding between LDH hydroxyl groups and PEI amines, a critical factor in stabilizing the composite interface.

**Figure 3:**
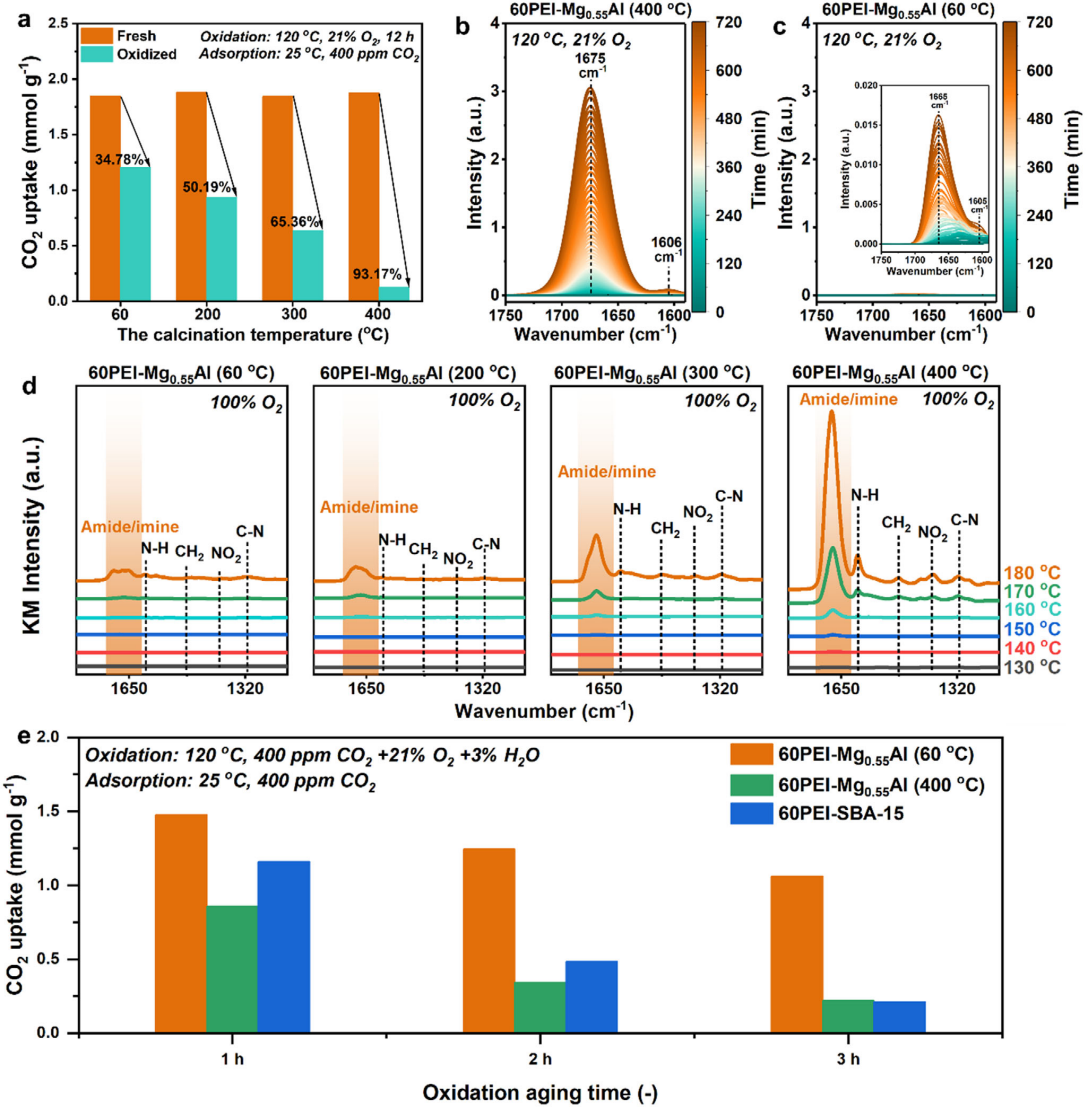
a) CO_2_ adsorption capacities of 60PEI‐Mg_0.55_Al (*y*) (*y* = 60, 200, 300, and 400 °C, respectively) before and after oxidative aging at 120 °C in 21% O_2_ for 12 h, measured at 25 °C under 400 ppm CO_2_/N_2_; b) in situ diffuse reflectance infrared Fourier transform spectroscopy (DRIFTS) spectra of 60PEI‐Mg_0.55_Al (400 °C) and c) 60PEI‐Mg_0.55_Al (60 °C) during exposure to 21% O_2_ at 120 °C for 12 h; d) in situ DRIFTS spectra of 60PEI‐Mg_0.55_Al (*y*) adsorbents recorded under 100% O_2_ while the temperature was continuously increased from 80 to 180 °C; e) CO_2_ uptakes of 60PEI‐Mg_0.55_Al (60 °C), 60PEI‐Mg_0.55_Al (400 °C) and 60PEI‐SBA‐15 after aging at 120 °C under 400 ppm CO_2_ + 21% O_2_ + 3% H_2_O, measured at 25 °C under 400 ppm CO_2_/N_2_.

**Figure** **3**a compares the CO_2_ adsorption capacities of 60PEI‐Mg_0.55_Al (*y*) adsorbents (*y* = 60, 200, 300, and 400 °C) before and after oxidative aging. Aging experiments were conducted in a quartz CSTR under 21% O_2_ at 120 °C for 12 h (40 mL min^−1^ flow rate), preceded by N_2_ pretreatment (120 °C, 1 h). Post‐aging CO_2_ uptake was quantified via TGA under 400 ppm CO_2_ at 25 °C for 180 min after identical N_2_ pretreatment.

A pronounced calcination‐dependent degradation trend emerged. The 60PEI‐Mg_0.55_Al (60 °C) adsorbent retained 65.2% of its initial capacity (1.85 to 1.21 mmol g^−1^, 34.8% loss), whereas higher calcination temperatures exacerbated oxidative degradation: 200 °C (50.2% loss, 1.88 to 0.94 mmol g^−1^), 300 °C (65.4% loss, 1.85 to 0.64 mmol g^−1^), and 400 °C (93.2% loss, 1.88 to 0.13 mmol g^−1^). This inverse correlation underscores the critical role of surface hydroxyl groups in stabilizing the composite framework. Thermal removal of these moieties at elevated temperatures likely disrupts LDH‐PEI hydrogen bonding, destabilizing the structure and accelerating amine degradation under oxidative conditions.

To probe degradation mechanisms, in situ diffuse reflectance infrared Fourier transform spectroscopy (DRIFTS) spectra were acquired during oxidative aging. Figure 3b,c reveal distinct spectral changes for 60PEI‐Mg_0.55_Al (400 °C) and 60PEI‐Mg_0.55_Al (60 °C) under 21% O_2_ at 120 °C. For the 400 °C sample, a prominent peak at 1675 cm^−1^ (C═O/C═N stretching of imide/amide) intensified over time, accompanied by a smaller peak at 1606 cm^−1^ (N─H bending of newly formed primary amines), confirming oxidative cleavage of amine groups.^[^ ^29^ ^]^ In contrast, these degradation signatures were markedly suppressed in the 60 °C sample, aligning with its superior CO_2_ retention. Figure 3d further illustrates calcination‐dependent degradation using DRIFTS spectra under 100% O_2_ (80–180 °C). Increasing calcination temperatures amplified C═O/C═N and N─H band intensities, corroborating progressive oxidative damage. This trend supports the hypothesis that surface hydroxyl groups on uncalcined LDH stabilize PEI via hydrogen bonding, mitigating oxidative chain scission.

To assess real‐world relevance, simulated air oxidation tests (400 ppm CO_2_ + 21% O_2_ + 3% H_2_O, 120 °C) were conducted. Figure 3e demonstrates that 60PEI‐Mg_0.55_Al (60 °C) retained 1.06 mmol g^−1^ CO_2_ uptake after 3 h, while the 400 °C counterpart degraded rapidly (0.22 mmol g^−1^). Notably, 60PEI‐SBA‐15, a silica‐supported benchmark, exhibited near‐complete capacity loss (0.21 mmol g^−1^) under identical conditions.

Our recent studies have suggested that Si─OH‐containing supports, such as SBA‐15, exhibit excellent oxidative stability under high CO_2_ flue gas conditions (10% CO_2_ + 5% O_2_ + 3% H_2_O), whereas PEI supported on substrates containing Al–OH surface groups suffers from severe oxidative degradation.^[^ ^15^ ^]^ This divergence stems from CO_2_ concentration effects: Under high‐CO_2_ flue gas (10–20%), surface Si–OH groups enhance PEI flexibility, promoting carbamate formation that inhibits oxidation. In contrast, for PEI‐LDH materials, the introduction of concentrated CO_2_ disrupts the protective hydrogen bonding network between PEI and Al‐OH, rendering the PEI chains more susceptible to oxidative exposure. Conversely, under DAC‐relevant low CO_2_ levels, hydrogen bonding between LDH hydroxyls and PEI dominates stabilization, outweighing CO_2_‐mediated destabilization observed in Al–OH systems. These findings collectively establish that uncalcined AMO‐LDHs provide a robust scaffold for PEI in DAC applications, leveraging hydroxyl‐mediated stabilization to achieve exceptional oxidative resistance under ambient air conditions.

### 2.5 Impact of Surface Hydroxyl Content on the Oxidative Stability of PEI‐LDH Adsorbents

**Figure 4:**
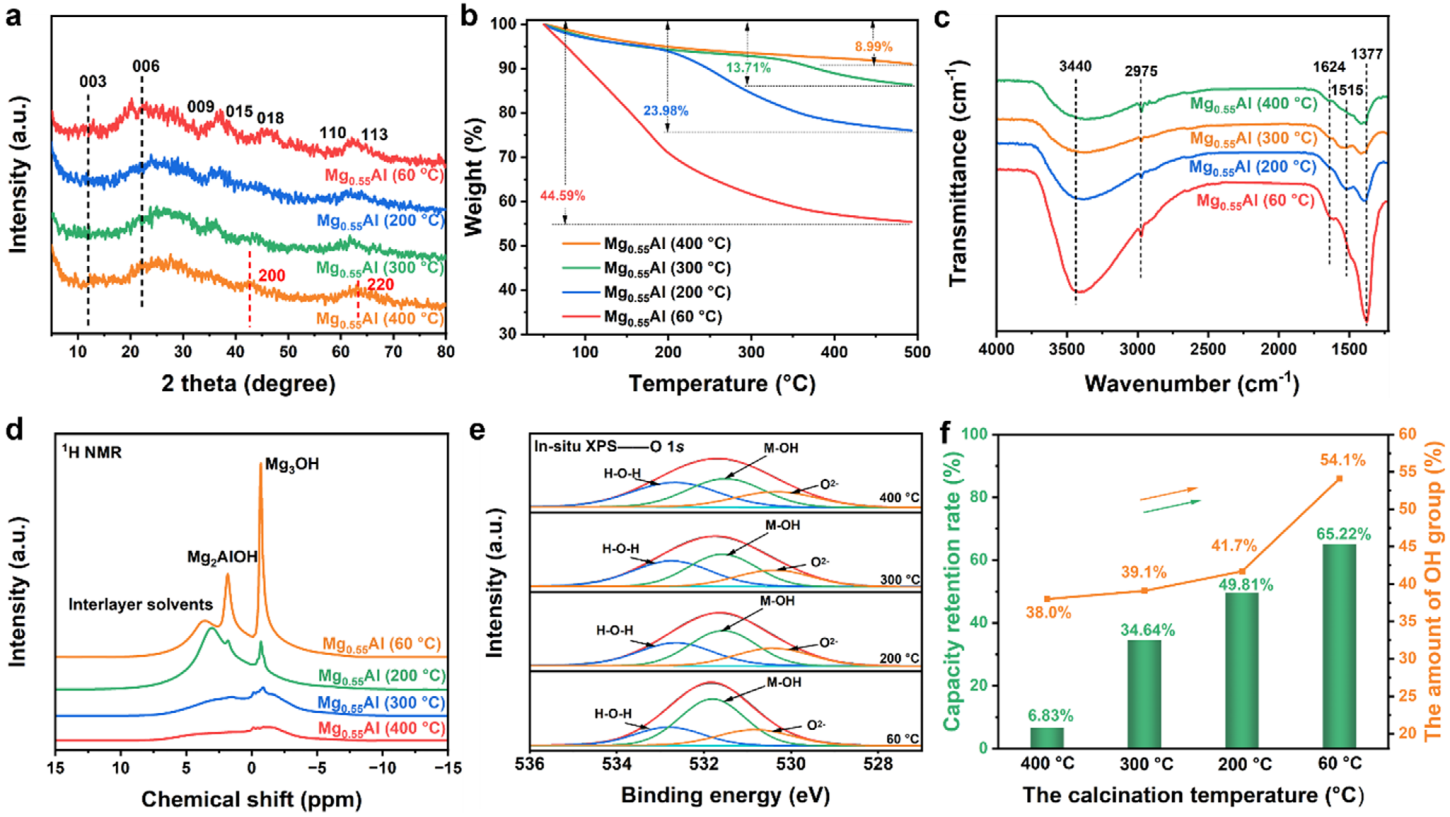
a) X‐ray diffraction patterns of Mg_0.55_Al (*y*) samples; b) Thermogravimetric analysis of Mg_0.55_Al (*y*) samples; c) Fourier transform infrared spectra of Mg_0.55_Al (*y*) samples; d) ^1^H solid‐state nuclear magnetic resonance spectra of Mg_0.55_Al (y) samples; e) In situ O 1s X‐ray photoelectron spectra of Mg_0.55_Al (60 °C) sample under N_2_ while continuously increasing the temperature from 60 to 400 °C; f) the relationship between the relative OH content in Mg_0.55_Al (*y*) samples and the capacity retention rate of 60PEI‐Mg_0.55_Al (*y*) samples after oxidative aging at 120 °C under 21% O_2_ for 12 h.

The structural evolution of Mg_0.55_Al‐CO_3_ LDH under calcination was systematically investigated to elucidate its impact on hydroxyl content and oxidative resistance. **Figure** **4**a shows the X‐ray diffraction (XRD) patterns, demonstrating a progressive transition from crystalline LDH to amorphous MMO with increasing calcination temperature (60–400 °C). The uncalcined sample (60 °C) retains the characteristic LDH Bragg reflections, (003), (006), (009), (015), (018), (110), and (113), while treatment at higher temperatures induces Bragg peak attenuation and shifting (e.g., (003) and (006) reflections shift to higher 2θ values), signifying interlayer carbonate decomposition and lattice contraction.^[^ ^21^, ^30^ ^]^ At 400 °C, the LDH structure is nearly completely lost, and weak MgO‐like (200) and (220) reflections emerge, confirming full MMO formation.^[^ ^17^, ^31^ ^]^

Thermal analysis (Figure 4b, TGA) reveals calcination‐dependent mass loss profiles. The 60 °C sample exhibits 44.59% weight loss, dominated by interlayer solvent desorption (200 °C) and dehydroxylation/carbonate decomposition (200–500 °C).^[^ ^30c^ ^]^ The decreases to 8.99% for the 400 °C sample, consistent with complete structural dehydration. FTIR spectra (Figure 4c) corroborate these trends: intensity reductions in O─H (3440 cm^−1^), H_2_O bending (1624 cm^−1^), and carbonate (1377 cm^−1^) modes confirm progressive hydroxyl and interlayer species removal.^[^ ^32^ ^]^

^1^H solid‐state nuclear magnetic resonance (NMR) spectra (Figure 4d) further resolves hydroxyl dynamics. The fresh LDH (60 °C) displays three distinct proton environments: Mg_3_OH (0.69 ppm), Mg_2_AlOH (1.82 ppm), and interlayer H_2_O (3.57 ppm).^[^ ^33^ ^]^ Calcination to 200 °C diminishes Mg_2_AlOH intensity while shifting its resonance to 3.04 ppm, reflecting altered coordination environments. Notably, the intensity of the ^1^H NMR signals gradually decreases with increasing treatment temperature, which aligns with TGA‐derived dehydroxylation trends.

To further explore the relationship between OH content and antioxidant capacity, surface hydroxyl quantification was conducted using in situ heating coupled with X‐ray photoelectron spectroscopy (XPS) analysis. The O 1s peak of Mg_0.55_Al (*y*) samples was deconvoluted into three components at binding energies of 532.8, 531.8, and 530.82 eV, corresponding to adsorbed water, hydroxyl groups, and M─O (M = Mg or Al), respectively (Figure 4e).^[^ ^34^ ^]^ The relative content of OH groups decreased significantly with increasing temperature: from 54.1% at 60 °C to 41.7% at 200 °C, and further to 39.1% and 38.0% at 300  and 400 °C, respectively, reflecting the dehydroxylation process. Critically, Figure 4f establishes a linear correlation between hydroxyl retention and oxidative stability for 60PEI‐Mg_0.55_Al (*y*) composites aged under 21% O_2_ (120 °C, 12 h). Samples with higher residual OH groups (e.g., 60 °C: 54.1% OH, 65.2% capacity retention) exhibit superior oxidative resistance compared to hydroxyl‐depleted counterparts (400 °C: 38.0% OH, 6.8% retention).

These multimodal analyses conclusively demonstrate that LDH calcination erodes surface hydroxyl populations, disrupting hydrogen‐bonding networks critical for PEI stabilization. Preserving hydroxyl‐rich LDH architectures thus emerges as a key strategy to mitigate oxidative degradation in DAC‐oriented adsorbents.

### 2.6 Impact of Surface Hydroxyl Groups on Water Vapor Co‐Adsorption Suppression: A Comparison of PEI‐LDH and PEI‐MMO Systems

Water vapor co‐adsorption critically impacts CO_2_ capture efficiency and regeneration energy demands in amine‐functionalized adsorbents.^[^ ^35^ ^]^ To evaluate the intrinsic hydrophilicity of the sorbent, we first measured water vapor adsorption–desorption isotherms at 25 °C (Figure S5, Supporting Information). The 60PEI‐Mg_0.55_Al (60 °C) composite exhibited moderate water uptake (8.79 mmol g^−1^ at 30% RH and 20.71  mmol g^−1^ at 80% RH), lower than hyper‐hygroscopic sorbents such as MOF‐303, as reported in literature (≈27.78 mmol g^−1^ at 30% RH).^[^ ^36^ ^]^ This restrained uptake suggests a limited number of accessible hydrophilic sites, likely due to hydrogen bonding between LDH hydroxyl groups and PEI chains.

**Figure 5:**
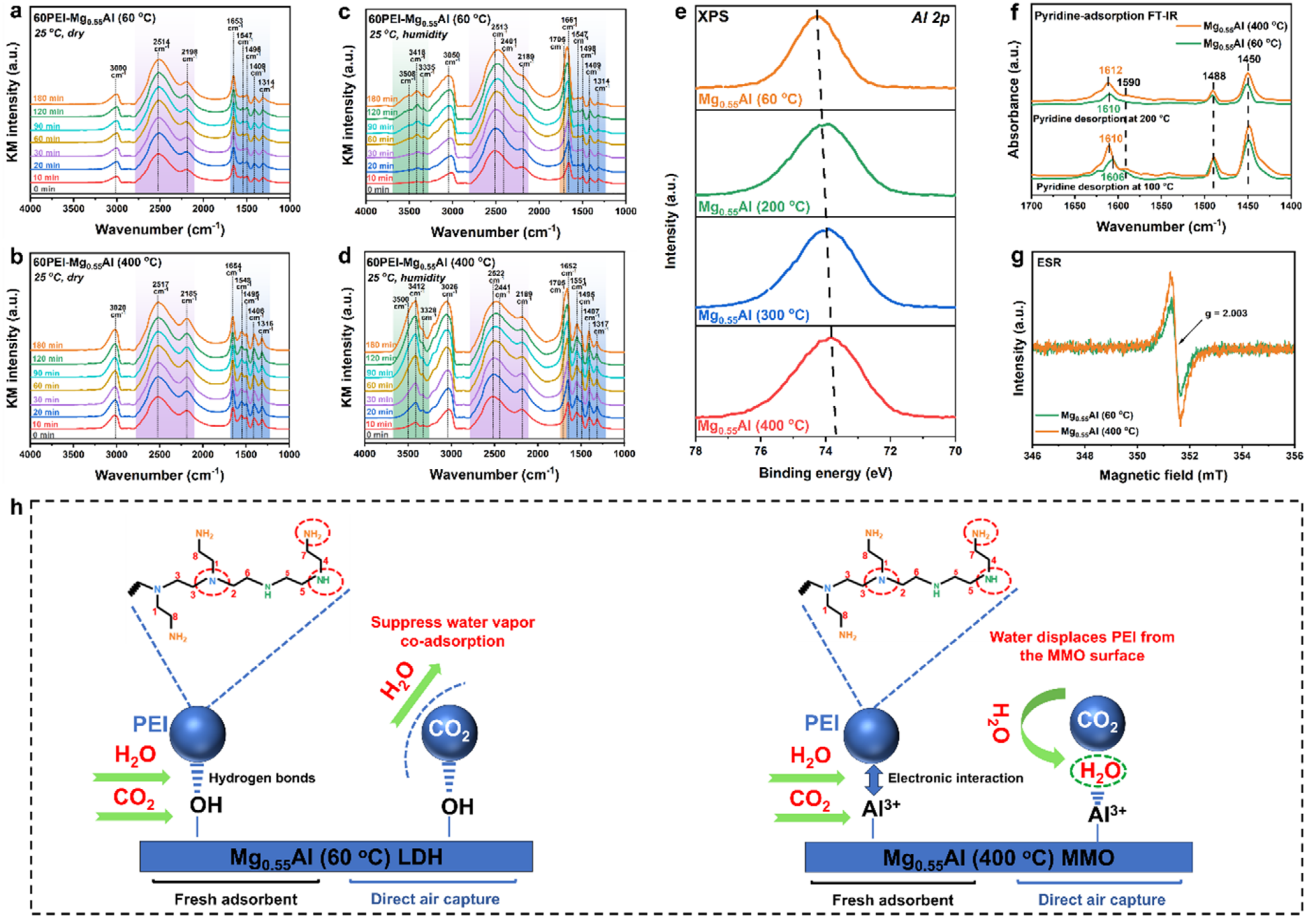
In situ diffuse reflectance infrared Fourier transform spectra of CO_2_ adsorption on 60PEI‐Mg_0.55_Al (*y*) under different conditions: a) 60PEI‐Mg_0.55_Al (60 °C) and b) 60PEI‐Mg_0.55_Al (400 °C) at 25 °C under dry conditions (400 ppm CO_2_/N_2_); c) 60PEI‐Mg_0.55_Al (60 °C) and d) 60PEI‐Mg_0.55_Al (400 °C) at 25 °C under humid conditions (400 ppm CO_2_/N_2_ + 3% H_2_O). e) X‐ray photoelectron spectra of Al 2p for Mg_0.55_Al (*y*); f) pyridine‐adsorption Fourier transform infrared spectra of Mg_0.55_Al (60 °C) and Mg_0.55_Al (400 °C); g) Electron spin resonance spectra of Mg_0.55_Al (60 °C) and Mg_0.55_Al (400 °C); h) schematic illustration of the suppression of water vapor co‐adsorption in PEI‐modified LDH and PEI‐modified MMO during direct air capture of CO_2_.

To elucidate the role of surface hydroxyl groups in mitigating this challenge, in situ DRIFTS studies were conducted on 60 wt.% PEI‐loaded Mg_0.55_Al materials (treated at 60 vs 400 °C) under dry and humid (3% H_2_O) conditions with 400 ppm CO_2_/N_2_. Under dry conditions (**Figure** **5**a,b), the CO_2_ adsorption process revealed several characteristic peaks: i) A series of bands in the 1200–1700 cm^−1^ region, indicative of ammonium carbamate ion pair formation. Specifically, the peak at 1653 cm^−1^ corresponds to the N─H deformation vibration of ammonium ions (RNH_3_^+^), while the peaks at 1547 and 1496 cm^−1^ are assigned to the C═O stretching vibrations in carbamate ions. Additionally, peaks at 1409 and 1314 cm^−1^ correspond to C─N stretching and NCOO^−^ deformation vibrations, respectively. ii) Broad bands centered at 2514 and 2198 cm^−1^, spanning the 2100–2800 cm^−1^ range, arise from hydrogen bonding interactions between ammonium ions (NH_3_^+^/NH_2_^+^) and carbamate ions (COO^−^). iii) A broad band centered at 3000 cm^−1^ in the 2900–3300 cm^−1^ range corresponds to hydrogen bonding interactions between ammonium ions and amine groups.^[^ ^37^ ^]^

Upon introducing water vapor into the system (Figure 5c,d), the IR spectra exhibited significant changes: i) A new water band appeared at 3508 cm^−1^, accompanied by the formation of hydrogen‐bonded water bands at 3418 and 3335 cm^−1^ in the 3000–3400 cm^−1^ region.^[^ ^15^ ^]^ ii) The 2100–2800 cm^−1^ range showed a pronounced enhancement of the broad IR band, particularly corresponding to the formation of hydronium carbamate (‐NCOO‐···H−OH_2_^+^) at 2401 cm^−1^.^[^ ^38^ ^]^ iii) The 1200–1700 cm^−1^ region, indicative of ammonium carbamate ion pairs, displayed increased intensity, accompanied by the formation of carbamic acid species, evidenced by a new peak at 1705 cm^−1^.

These changes were observed in both the LDH and MMO systems, highlighting the role of water vapor in facilitating the formation of ammonium carbamate species and enhancing the amines efficiency. However, the water co‐adsorption significantly increases the regeneration heat and energy consumption, making the effective suppression of water co‐adsorption crucial for the large‐scale deployment of DAC systems.

Interestingly, the ability to suppress water vapor co‐adsorption varies between systems. Compared to 60PEI‐Mg_0.55_Al (400 °C), 60PEI‐Mg_0.55_Al (60 °C) more effectively suppresses water vapor co‐adsorption. This is reflected in the significantly weaker intensity of the hydrogen‐bonded water bands (3300–3400 cm^−1^) and the water band at 3561 cm^−1^ in the IR spectra of the PEI‐LDH system, compared to the PEI‐MMO system.^[^ ^39^ ^]^

To further clarify the sequential adsorption behavior, time‐resolved in situ DRIFTS measurements were performed during the initial 10 min under humid DAC conditions (400 ppm CO_2_, 3% H_2_O, 25 °C). Both PEI‐LDH and PEI‐MMO samples exhibited immediate appearance of CO_2_‐related bands (1200–1700 cm^−1^ region) within the first minute, indicating preferential CO_2_ adsorption.^[^ ^40^ ^]^ In contrast, water‐related bands (3508, 3418, and 3335 cm^−1^) appeared later, with a delayed onset around the 6th min for PEI‐LDH and earlier around the 3rd min for PEI‐MMO (Figure S6, Supporting Information). These results confirm that CO_2_ adsorption precedes water uptake and highlight the superior ability of hydroxyl‐rich LDH to suppress water co‐adsorption. This suggests that the PEI‐LDH system maintains superior CO_2_ adsorption capacity while effectively minimizing water vapor co‐adsorption.

To elucidate the distinct water adsorption resistance mechanisms of PEI supported on LDH versus MMO, we performed systematic structural analyses. XPS spectral deconvolution of the Al 2*p* core level (Figure 5e) tracked the thermally induced coordination changes. A 0.4 eV negative binding energy shift occurred with the increase in calcination temperature (60 to 400 °C), aligning with the gradual transition of Al^3+^ from octahedral to tetrahedral geometry.^[^ ^41^ ^]^ This structural evolution correlated with hydroxyl group depletion (estimated via in situ O 1s spectral analysis) and the concomitant generation of electron‐deficient Lewis acid sites.^[^ ^17^ ^]^ Supporting this interpretation, pyridine‐FTIR spectra (Figure 5f) exhibited stronger adsorption bands at 1612, 1590, 1488, and 1450 cm^−1^ for 400 °C‐MMO compared to LDH, unambiguously confirming the abundance of coordinatively unsaturated Al^3+^ centers.^[^ ^7^, ^42^ ^]^ Quantitative analysis based on desorption peak intensities ≈1450 cm^−1^ further supported this observation. The Mg_0.55_Al MMO (400 °C) exhibited 31% higher Lewis acid site density (111.05 µmol g^−1^) than the LDH material (84.75 µmol g^−1^) at 100 °C. This disparity was sustained at elevated temperatures (200 °C), with MMO maintaining 58% higher acid site concentration (53.61 vs 33.98 µmol g^−1^) (Figure S7, Supporting Information). The enhanced Lewis acidity results from the thermally induced reduction in Al^3+^ coordination number, transitioning from a six‐fold (octahedral) to a four‐fold (tetrahedral) geometry. This structural transformation increases surface electron deficiency by reducing metal‐ligand shielding effects, thereby enhancing the electrophilic character of Al^3+^ centers.^[^ ^41^ ^]^

Electron spin resonance spectroscopy quantitatively resolved oxygen vacancy dynamics (Figure 5g). Both Mg_0.55_Al phases showed characteristic signals at g ≈ 2.003, indicative of trapped electrons at oxygen vacancies.^[^ ^12^ ^]^ Strikingly, the MMO phase displayed a marked intensity enhancement over LDH, directly linking thermal activation to the formation of oxygen vacancies in the Al─O─Al structure and subsequent proliferation of Lewis acid sites.

These structural disparities dictate divergent polymer‐support interactions. Previous work from our group has established that amine groups bind to MMO's Lewis acid sites through electron transfer.^[^ ^12^ ^]^ However, this work reveals that water vapor may competitively displace PEI from these sites.^[^ ^43^ ^]^ The collapse of the layered structure and the loss of hydroxyl groups in PEI‐MMO lead to uneven PEI distribution. This structural degradation increases the accessibility of pores to water molecules, allowing rapid migration of water into the open pores and subsequent adsorption onto the exposed, highly active Lewis acid sites (e.g., Al^3+^) on the MMO surface. This results in displacement or coverage of the amine groups by water molecules, further exacerbating the co‐adsorption phenomenon. In contrast, compared to PEI‐MMO, the enhanced ability of PEI‐LDH to suppress water vapor co‐adsorption can be attributed to its unique layered structure and abundant surface hydroxyl groups. Strong hydrogen bonding interactions between the amine groups and surface hydroxyls promote a more uniform distribution of PEI and effectively anchor the amine groups, preventing their displacement by water molecules (Figure 5h).

These findings underscore that hydroxyl‐rich LDH scaffolds not only preserve CO_2_ capacity in humid conditions but also minimize parasitic water uptake—a dual advantage critical for energy‐efficient DAC deployment.

## 3 Conclusion

This work establishes a scalable, low‐carbon synthesis route for PEI‐functionalized Mg_0.55_Al AMO LDHs, offering a sustainable pathway for DAC deployment. Three key advances position PEI@LDH composites as competitive candidates for industrial‐scale CO_2_ removal: i) The Mg_0.55_Al LDH precursor was synthesized via a low‐cost, scalable AMOST process that avoids high‐temperature calcination, significantly reducing both energy consumption and environmental impact compared to conventional methods. This approach supports the feasibility of large‐scale production of PEI@LDH adsorbents, with a production cost of ≈$7‐8 per kilogram for AMO LDH.^[^ ^32b^ ^]^ ii) The impregnation of PEI onto the Mg_0.55_Al LDH support was conducted using a recyclable, environmentally friendly methanol solvent, ensuring minimal ecological footprint and cost efficiency. iii) The PEI@LDH adsorbents achieve a CO_2_ uptake of 3.92 mmol g^−1^ under simulated air and retain >90% of their initial CO_2_ capacity over 20 adsorption‐regeneration cycles. This excellent performance stems from AMOST induced mesopore engineering as well as hydroxyl‐mediated PEI anchoring, which suppresses oxidative degradation and minimizes water co‐adsorption.

These advances address critical barriers in DAC commercialization: energy‐intensive material synthesis, oxidative degradation, and humidity sensitivity. This environmentally friendly and cost‐effective CO_2_ capture system holds substantial promise for mitigating CO_2_ emissions, particularly in DAC applications. The ability of PEI@LDH adsorbents to perform effectively under diverse environmental conditions—such as exposure to moisture and trace gases—further supports their integration into real‐world DAC systems.

Moreover, the combined use of a scalable AMOST synthesis method for Mg_0.55_Al LDHs and a mature PEI impregnation process utilizing recyclable solvents demonstrates strong feasibility for large‐scale manufacturing. Current efforts are focused on developing pelletization techniques to produce mechanically robust adsorbent forms suitable for industrial fixed‐bed reactors. Inspired by recent advances in molecular‐level design strategies, such as those demonstrated in stretchable hydrogel systems,^[^ ^44^ ^]^ the potential formation of hydrogen bonding networks and polymer chain entanglements in pelletized PEI@LDH composites may offer enhanced flexibility and mechanical stability for practical deployment.

Future efforts will focus on i) scaling AMO‐LDH production to multi‐ton/month capacity, ii) optimizing amine loading and exploring molecular modifications (e.g., epoxide treatment,^[^ ^45^ ^]^ alternative polymers,^[^ ^46^ ^]^ additives,^[^ ^47^ ^]^ hybrid amine systems^[^ ^8b^ ^]^) to improve long‐term stability and humidity resilience, and iii) integrating structured adsorbents with a low‐grade heat steam‐assisted regeneration system (<100 °C). By bridging material innovation with process engineering, this work provides a viable roadmap for gigaton‐scale DAC deployment.

## Conflict of Interest

The authors declare no conflict of interest.

## Supporting information



Supporting Information

## Data Availability

The data that support the findings of this study are available in the supplementary material of this article.
