# Supplementary material for: Innovative Design of PEI‐Modified AMO‐Layered Double Hydroxide for Efficient and Stable Direct Air Capture of CO2
Source: Adv Sci (Weinh). 2025 Jun 30;12(37):e07756. doi: 10.1002/advs.202507756 (PMC12499402; doi:10.1002/advs.202507756)
Supplement: Supplementary file 1 — Supporting Information [file ADVS-12-e07756-s001.docx]

Supporting Information

**Efficient and Stable Direct Air Capture of CO_2_ using Polyethyleneimine-Modified Layered Double Hydroxide: Key Roles of Porosity and Surface Hydroxyl Groups**

*Meng Zhao^1^, Liang Huang^1^, Yanshan Gao^1,2^*, Ziling Wang^1^, Xuancan Zhu^3^, Qiang Wang^1,2^*, Dermot O’Hare^4^*

**Experimental Section/Method**

*Adsorbent Preparation*: Mg_0.55_Al LDH was synthesized via the AMOST method.^[1]^ Briefly, 50 mL of a solution containing 4.549 g of Mg(NO_3_)_2_·6H_2_O and 12.101 g of Al(NO_3_)_3_·9H_2_O was slowly added to an equal volume of a solution containing 2.650 g of Na_2_CO_3_, with vigorous stirring. The pH was adjusted to 10 by the gradual addition of a 4 M NaOH solution. The mixture was aged at 60 °C for 12 hours, followed by filtration and washing with deionized water to remove residual ions and adjust the pH to 7. The solids were rinsed with ethanol, redispersed in ethanol, and stirred at 25 °C for 2 hours before filtration.The samples were dried under vacuum at 60 °C for 12 hours and then ground into fine powder. The Mg_0.55_Al-CO_3_ LDH was subsequently calcined at various temperatures in a tube furnace under N_2_ flow for 5 hours. The obtained samples were labeled as Mg_0.55_Al (*y*), where "*y*" corresponds to the calcination temperature in °C. Specifically, Mg_0.55_Al (60 °C) refers to the uncalcined Mg_0.55_Al LDH powder that was dried under vacuum at 60 °C but not calcined, whereas Mg_0.55_Al (200 °C), Mg_0.55_Al (300 °C), and Mg_0.55_Al (400 °C) denote samples calcined at 200, 300, and 400 °C, respectively, in a tube furnace under N_2_ flow for 5 hours.

The functionalization of PEI was carried out via the convenient impregnation method. A predetermined amount of branched polyethyleneimine (PEI, Shanghai Meryer Chemical Technology Co., Ltd, MW 600, 99%) was dissolved in 50 mL of methanol. To this solution, 0.25 g of Mg_0.55_Al (*y*) powder was added, and the mixture was stirred for 6 hours at 25 ^o^C to ensure uniform impregnation. The solvent was then removed by rotary evaporation, and the resulting composite was dried under vacuum at 60 °C for 12 hours. The final adsorbents were denoted as *x*PEI-Mg_0.55_Al (*y*), where "*x*" represents the weight ratio of PEI in the composite material and "*y*" refers to the calcination temperature of the Mg_0.55_Al substrate. To investigate the porosity enhancement induced by AMOST treatment, a conventional Mg_0.55_Al-water (60 °C) LDH was synthesized via the co-precipitation method without AMOST treatment. Here, Mg_0.55_Al-water (60 °C) indicates LDH synthesized via co-precipitation, followed by water washing and drying under vacuum at 60 °C. The washed precipitate was then used to support PEI, yielding the adsorbent denoted as *x*PEI-Mg_0.55_Al-water (60 °C). For comparison, SBA-15 (Nanjing XFNANO Materials Tech Co., Ltd) was employed as a silica-supported benchmark for PEI loading, and these samples were designated as *x*PEI-SBA-15, where "*x*" denotes the weight ratio of PEI in the adsorbents.

*Adsorbent Characterization*: The structural and chemical properties of the adsorbents were systematically characterized using a suite of analytical techniques. Crystallinity and phase composition were analyzed by X-ray diffraction (XRD), while surface morphology and elemental distribution were examined via scanning/transmission electron microscopy (SEM/TEM) coupled with energy-dispersive spectroscopy (EDS). Nitrogen physisorption measurements were performed to determine the specific surface area, pore volume, and pore size distribution, applying the Brunauer-Emmett-Teller (BET) and Barrett-Joyner-Halenda (BJH) models. X-ray diffraction (XRD) analysis was conducted to examine the crystallinity of the materials. XRD patterns were recorded using a Shimadzu XRD-7000 diffractometer with Cu Kα radiation in the 2θ range of 5~80° at a scanning speed of 5° min^−1^. The surface morphology of the adsorbents was examined using scanning electron microscopy (SEM) on a Hitachi SU8010 microscope at an acceleration voltage of 10 kV. The samples were coated with a thin layer of gold to enhance imaging. Elemental composition was determined by energy dispersive spectroscopy (EDS) coupled with the SEM, using a HORIBA-EMX system. Transmission electron microscopy (TEM) was performed using a FEI Tecnai G2 F20 TEM (FEI Company, USA), operated at an accelerating voltage of 200 kV. The point resolution was 0.24 nm, STEM resolution was 0.2 nm, and the information resolution was ≤ 0.14 nm. The specific surface area, average pore diameter, and pore volume of the synthesized adsorbents were determined using nitrogen adsorption-desorption isotherms at 77 K, measured with a Builder SSA-7000 apparatus. The specific surface area and average pore diameter were calculated using the Brunauer-Emmett-Teller (BET) method. Pore volume was analyzed using the Barrett-Joyner-Halenda (BJH) method, based on the desorption branch of the isotherm.

Surface functional groups and thermal stability were assessed using Fourier transform infrared (FTIR) spectroscopy and thermogravimetric analysis (TGA), respectively. Fourier transform infrared (FTIR) spectroscopy was employed to probe the surface functional groups of the adsorbents. FTIR spectra were recorded on a Nicolet IS10 spectrometer (Thermo Scientific, USA) in the range of 4000~400 cm^−1^ with a resolution of 4 cm^−1^. Thermogravimetric analysis (TG) was conducted to evaluate the thermal stability of the adsorbents. Measurements were performed using a TA55 thermogravimetric analyzer under a nitrogen flow of 40 mL min^−1^, with a heating rate of 10 °C min^−1^ from ambient temperature to 500 °C.

To probe surface chemistry, solid-state ^1^H nuclear magnetic resonance (^1^H NMR) and X-ray photoelectron spectroscopy (XPS) were employed. Acidic sites and oxygen vacancies were quantified using pyridine-adsorption FTIR (Py-FTIR) and electron spin resonance (ESR) spectroscopy. Proton nuclear magnetic resonance (^1^H NMR) experiments were carried out on a Bruker AVANCE III 600 MHz NMR spectrometer to study the structural evolution of surface hydroxyl groups in Mg_0.55_Al (*y*) samples subjected to varying calcination temperatures. Spectra were acquired at a 15 kHz operating frequency with 32 scans. X-ray photoelectron spectroscopy (XPS) was performed using a Thermo Fisher Scientific Escalab 250Xi instrument with monochromatic Al Kα radiation (1486.6 eV) to analyze the surface composition and chemical state of the adsorbents. The pyridine infrared adsorption spectra (Py-FTIR) were recorded using a Bruker Tensor 27 Fourier transform infrared spectrometer, with 32 scans and a resolution of 4 cm⁻^1^. For the test, approximately 14 mg of the sample was pressed into a 13 mm self-supported disk and placed in the *in-situ* cell of the spectrometer. The sample was evacuated to 10^−3^ Pa at 100°C for 2 hours to remove gas molecules from the surface. Subsequently, the sample was saturated with pyridine vapor at 40 °C for 30 minutes. Pyridine desorption was then performed at 100 °C and 200 °C, and spectra were recorded in the 1400~1700 cm^−1^ wavenumber range. Electron spin resonance (ESR) spectroscopy was performed at room temperature using a Bruker A300-10/12 spectrometer to characterize the oxygen vacancies in the samples. Approximately 10 mg of the powder sample was accurately weighed and loaded into a quartz ESR tube, which was then placed in the spectrometer for measurement. The spectra were recorded at a microwave frequency of 9.853 GHz with a microwave power of 20.03 mW.

*CO_2_ Adsorption Studies*: CO_2_ adsorption was performed using a TA55 thermogravimetric analyzer to assess the CO_2_ uptake of the adsorbents. Prior to adsorption, approximately 10 mg of the sample was pre-treated at 120 °C under a N_2_ flow (40 mL min⁻^1^) for 1 hour to remove any pre-adsorbed CO_2_ and H_2_O. The sample was then exposed to a 400 ppm CO_2_/N_2_ flow (40 mL min^−1^) at 25 °C for 180 minutes. Adsorption data were collected throughout the experiment to determine CO_2_ uptake.

For the regeneration tests, after CO_2_ adsorption, the sample was desorbed by heating to 120 °C for 15 minutes under a N_2_ flow (40 mL min^−1^). The adsorption-desorption cycle was repeated 20 times to evaluate the cyclic stability and reusability of the adsorbent.

CO_2_ breakthrough experiments were carried out in a continuous stirred tank reactor (CSTR) microreactor under simulated air conditions (400 ppm CO_2_, 21% O_2_, 3 vol% H_2_O). Approximately 0.1 g of the adsorbent was pre-treated at 120 °C under a 100 mL min^−1^ N_2_ flow for 60 minutes, followed by cooling to 25 °C. The adsorption test was performed at 25 °C with a 100 mL min^−1^ gas flow for 550 minutes. CO_2_ concentration at the reactor outlet was continuously monitored using a THA100S non-dispersive infrared analyzer. CO_2_ uptake was determined by applying **Equation 1** to the breakthrough experiments, with blank measurements subtracted to ensure accuracy.

$q=\frac{\int_{0}^{t} (C_{CO_{2},in}-C_{CO_{2},out})Q_{in}dt}{m}$ (1)

$q$ CO_2_ uptakes (mmol g^-1^)

$C_{CO_{2},in}$ CO_2_ concentration in the inlet stream (mmol m^-3^)

$C_{CO_{2},out}$ CO_2_ concentration in the outlet stream (mmol m^-3^)

$Q_{in}$ Flow rate of the inlet stream (m^3^ min^-1^)

$m$ Initial mass of the adsorbent (g)

*Oxidative Aging Experiments*: Oxidative aging experiments were performed using a home-made transient gas flow system. 0.1 g sample of the adsorbent was first pretreated in a quartz CSTR micro-reactor at 120 °C for 1 hour under a nitrogen flow of 40 mL min^−1^. Following the pre-treatment, the system was switched to an oxidative atmosphere, maintaining the temperature at 120 °C. The oxidative aging was carried out under two gas conditions: 21% O_2_, balanced N_2_ or a mixture of 400 ppm CO_2_, 21% O_2_, 3% H_2_O, balanced N_2_. Moisture in the inlet gas was generated by passing the gas stream through a scrubbing bottle immersed in a thermostatic water bath. The gas flow rate during both pre-treatment and oxidative aging stages was maintained at 40 mL min^−1^.

*In-situ DRIFTS Studies*: The *in-situ* DRIFTS experiments were conducted using a PerkinElmer Spectrum 3 FTIR spectrometer (4 cm^−1^ resolution) equipped with an MCT detector. Spectra were collected in the range of 4000~700 cm^−1^, with 128 scans per spectrum. Approximately 50 mg of the sample was loaded into a temperature-controlled *in-situ* DRIFTS reactor cell with a ZnSe window. Prior to each reaction, the sample was pretreated in a nitrogen flow (40 mL min^−1^) at 120 °C for 1 hour to remove any adsorbed species. During both the pre-treatment and adsorption/oxidation stages, the gas flow rate was maintained at 40 mL min^−1^. For experiments involving moisture, water vapor (3 vol% H_2_O) was introduced by passing the gas stream through a scrubbing bottle immersed in a thermostatic water bath.

**Table S1.** Textural properties of the LDH/MMO.

| Sample | Specific surface area  (m^2^ g^-1^) | Total pore volume  (cc g^-1^) | Average pore radius  (nm) |
| --- | --- | --- | --- |
| Mg_0.55_Al-water (60 ^o^C) | 188.49 | 0.36 | 2.81 |
| Mg_0.55_Al (60 ^o^C) | 358.43 | 1.64 | 8.07 |
| Mg_0.55_Al (400 ^o^C) | 377.74 | 1.88 | 8.82 |

**Ta****ble** **S2.** Comparison of specific surface area and pore volume of LDHs synthesized via AMOST and conventional methods.

| LDH composition | Interlayer anion | Synthesis method | Specific surface area  (m^2^ g^-1^) | Total pore volume  (cc g^-1^) | Ref. |
| --- | --- | --- | --- | --- | --- |
| MgAl | [B_4_O_5_(OH)_4_]^2-^ | Co-precipitation | 1.0 | 0.01 | ^[1a]^ |
| LiAl | CO_3_^2-^ | Co-precipitation | 84.11 | 0.57 | ^[2]^ |
| CuCoAl | CO_3_^2-^ | Co-precipitation | 87.3 | - | ^[3]^ |
| CuZnAl | CO_3_^2-^ | Co-precipitation | 48 | 0.2 | ^[4]^ |
| MgAl | CO_3_^2-^ | Co-precipitation | 114 | - | ^[5]^ |
| MgAl | CO_3_^2-^ | Co-precipitation + hydrothermal | 28 | - | ^[5]^ |
| MgAl | CO_3_^2-^ | Urea | 17 | - | ^[5]^ |
| MgAl | CO_3_^2-^ | Urea-microwave | 8 | - | ^[5]^ |
| MgAl | CO_3_^2-^ | Urea-hydrothermal | 6 | - | ^[5]^ |
| NiFe | CO_3_^2-^ | Hydrothermal | 57.116 ± 0.523 | 0.315 ± 0.004 | ^[6]^ |
| NiCo | CO_3_^2-^ | Hydrothermal | 12.1 | - | ^[7]^ |
| MnNiCo | CO_3_^2-^ | Pre-deposited AMO + hydrothermal | 24.9 | - | ^[7]^ |
| CaAl | NO_3_^-^ | In situ electrodeposition | 26 | - | ^[8]^ |
| CaAl | Cl^-^ | In situ electrodeposition | 37 | - | ^[8]^ |
| MgAl | [B_4_O_5_(OH)_4_]^2-^ | AMOST | 263.0 | 1.07 | ^[1a]^ |
| MgAl | CO_3_^2-^ | AMOST | 358.43 | 1.64 | This work |

**Table S3.** Textural properties of the PEI-modified LDH.

| Sample | Specific surface area  (m^2^ g^-1^) | Total pore volume  (cc g^-1^) | Average pore radius  (nm) |
| --- | --- | --- | --- |
| 33PEI-Mg_0.55_Al-water (60 ^o^C) | - | - | - |
| 33PEI-Mg_0.55_Al (60 ^o^C) | 157.75 | 0.90 | 10.12 |
| 50PEI-Mg_0.55_Al (60 ^o^C) | 30.19 | 0.20 | 12.7 |
| 60PEI-Mg_0.55_Al (60 ^o^C) | 11.06 | 0.07 | 10.79 |
| 67PEI-Mg_0.55_Al (60 ^o^C) | 6.02 | 0.03 | 8.91 |

**Table S4.** Comparison of CO_2_ uptakes for amine-modified LDH adsorbents at 25 °C. Abbreviations: 3-[2-(2-aminoethylamino)ethylamino]propyl-trimethoxysilane (TRI), (3-aminopropyl)triethoxysilane (APS), fast misxing with DMF treatment (FMDT).

| LDH composition | Amine | Synthesis method | Adsorption conditions | Capacity  [mmol g^-1^] | Ref. |
| --- | --- | --- | --- | --- | --- |
| MgAl | TRI | FMDT + grafting | Dry, 25 °C, 400 ppm CO_2_/N_2_ | 0.68 | ^[9]^ |
| MgAl | TRI | FMDT + in-situ grafting | Dry, 25 °C, 400 ppm CO_2_/N_2_ | 0.98 | ^[10]^ |
| MgAl | TRI | FMDT + in-situ grafting | 20% RH, 400 ppm CO_2_/N_2_ | 1.16 | ^[10]^ |
| MgAl | APS | Co-precipitation + grafting | Dry, 25 °C, pure CO_2_ | 1.55 | ^[11]^ |
| MgAl | PEI | AMOST + impregnation | 3% H_2_O, 25 °C,  400 ppm CO_2_/21% O_2_/N_2_ | 3.92 | This work |

**Table S5.** CO_2_ adsorption reproducibility and batch consistency of 60PEI-Mg_0.55_Al (60 °C) (400 ppm CO_2_/N_2_, 25 °C).

| Test Type | 1 | 2 | 3 | 4 | RSD (%) |
| --- | --- | --- | --- | --- | --- |
| Intra-batch (same sample) | 1.85 | 1.81 | 1.90 | 1.92 | 2.66 |
| Inter-batch (different batches) | 1.85 | 1.92 | 1.95 | 1.87 | 2.41 |

*
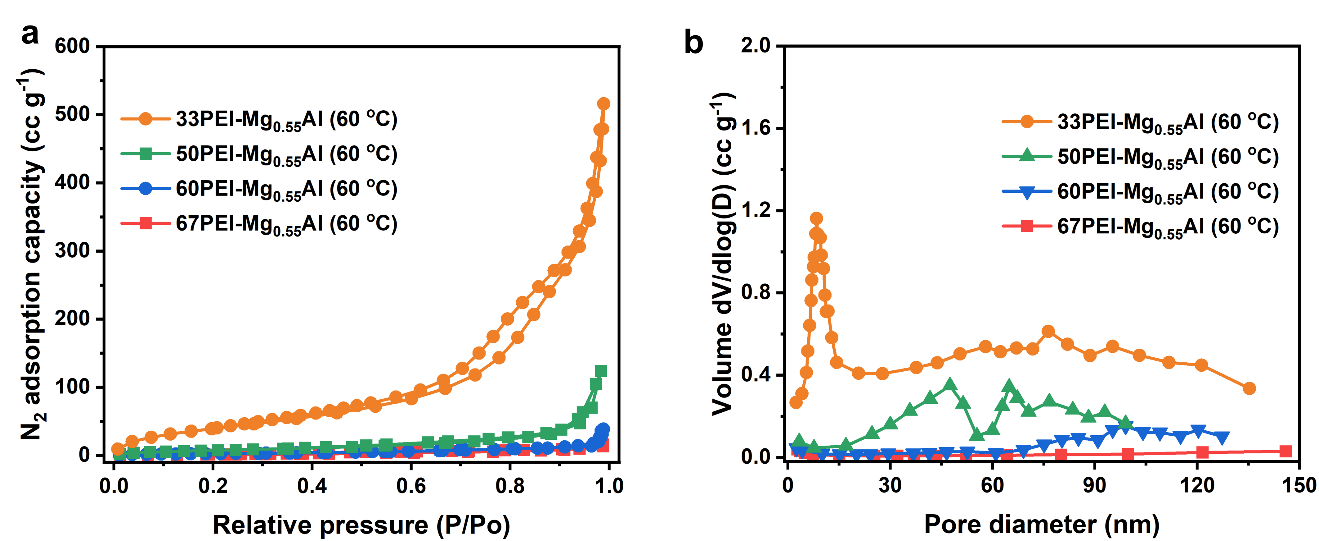
*

**Figure S1.** (a) N_2_ adsorption-desorption isotherms and (b) pore size distributions of *x*PEI-Mg_0.55_Al (60 ^o^C).

*
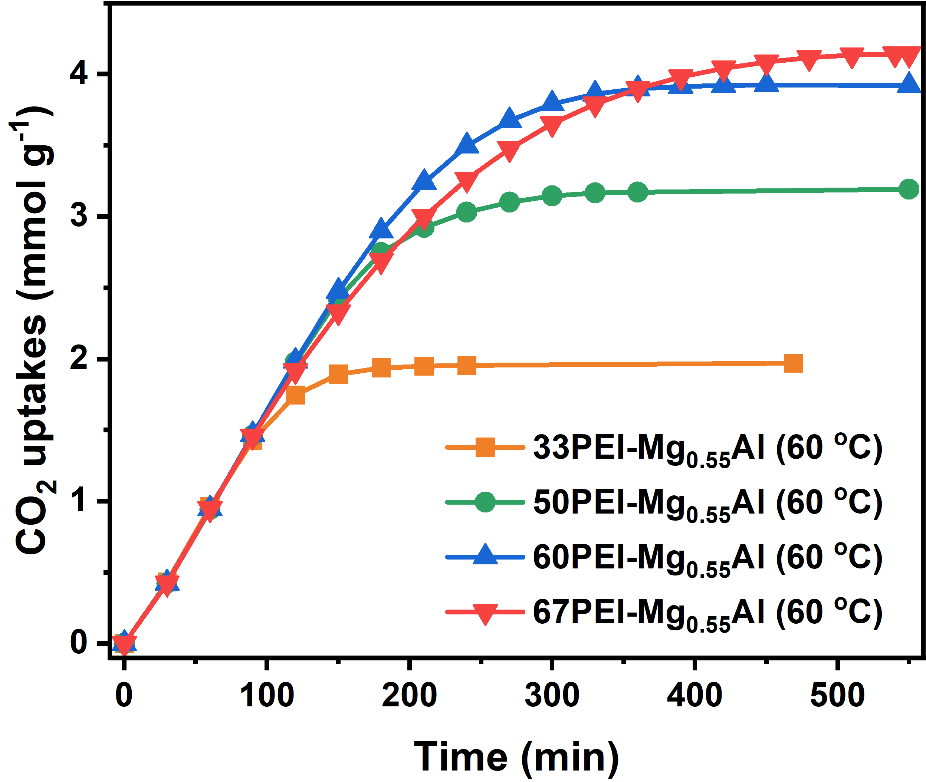
*

**Figure S2.** Time-resolved CO_2_ uptake profiles of *x*PEI-Mg_0.55_Al (60 ^o^C) recorded during CO_2_ breakthrough testing at 25 °C in a gas mixture of 400 ppm CO_2_, 21% O_2_, and 3% H_2_O.

*
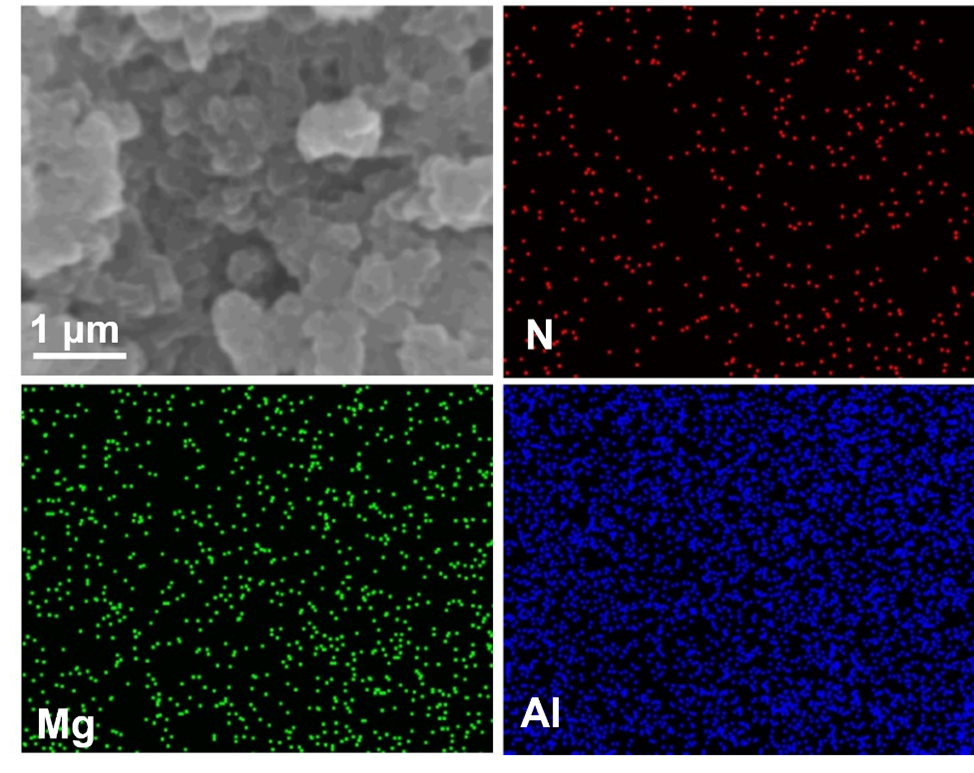
*

**Figure S3.** Energy dispersive X-ray spectroscopy (EDS) mapping of the regenerated 60PEI-Mg_0.55_Al (60 °C) sample.

*
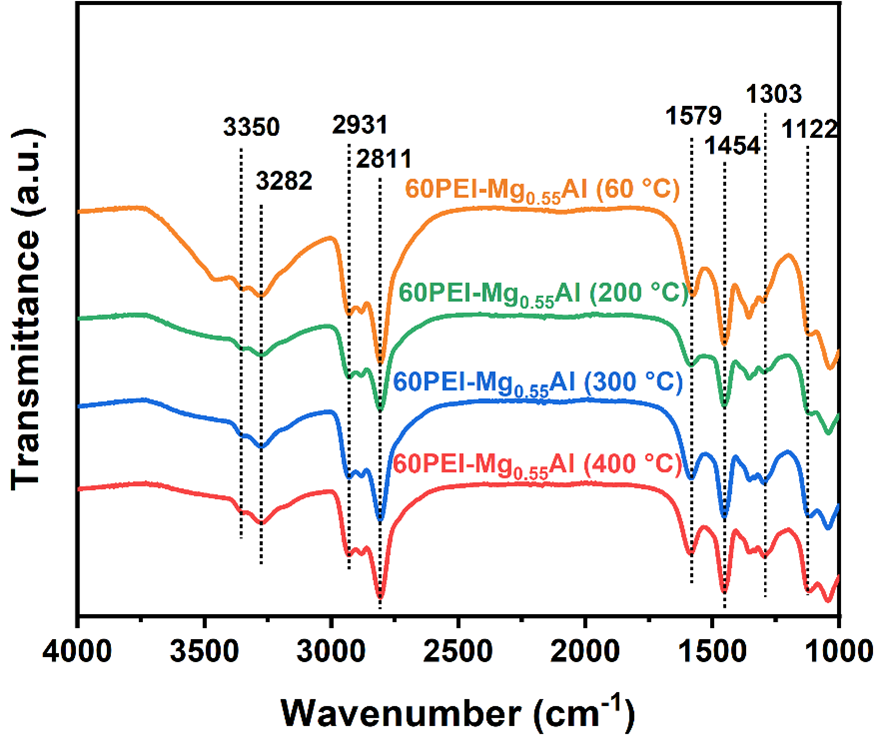
*

**Figure S4.** FT-IR spectra of the 60PEI-Mg_0.55_Al (*y*) (*y* = 60, 200, 300 and 400 °C).

*
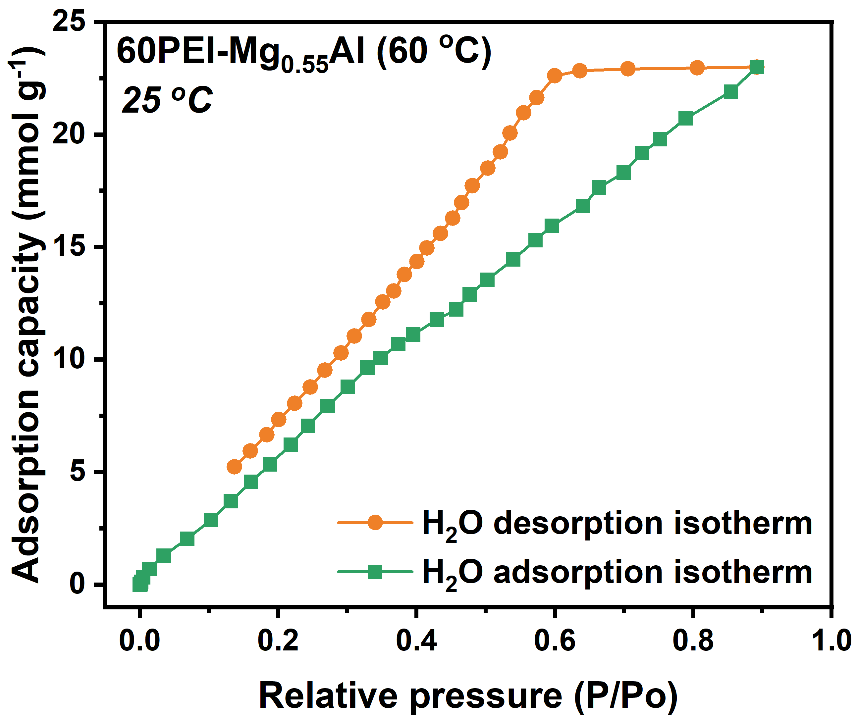
*

**Figure S5.** Water vapor adsorption-desorption isotherms of 60PEI-Mg_0.55_Al (60 °C) measured at 25 °C.

*
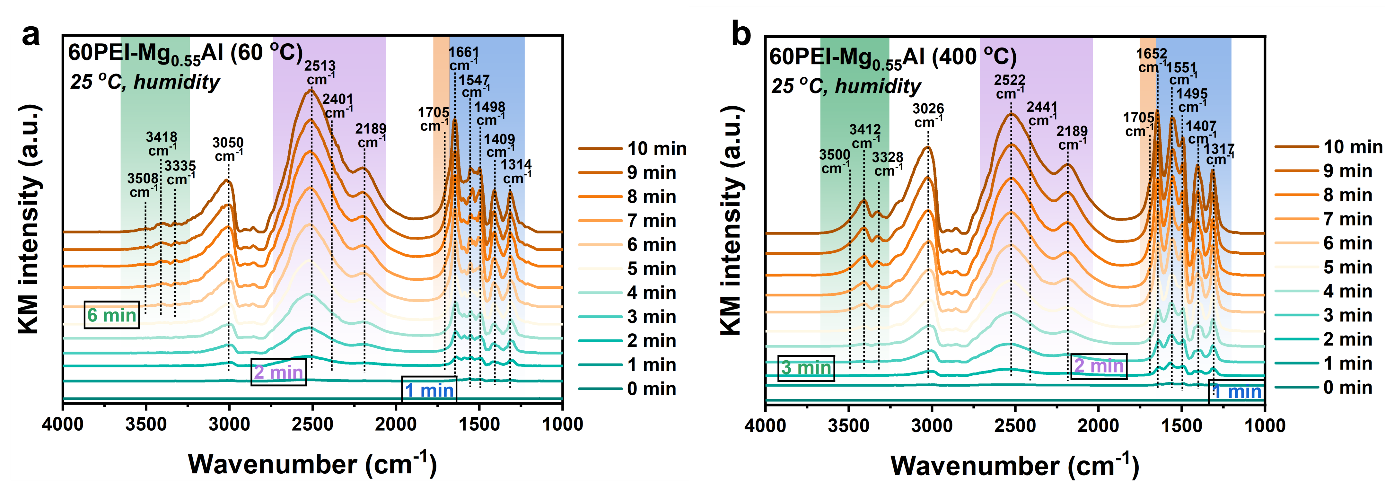
*

**Figure S6.** Time-resolved in-situ DRIFTS spectra of (a) 60PEI-Mg_0.55_Al (60 °C), and (b) 60PEI-Mg_0.55_Al (400 °C) under simulated humid DAC conditions (400 ppm CO_2_, 3% H_2_O, N_2_ balance, 25 °C) recorded at 1-minute intervals during the first 10 minutes of exposure.

*
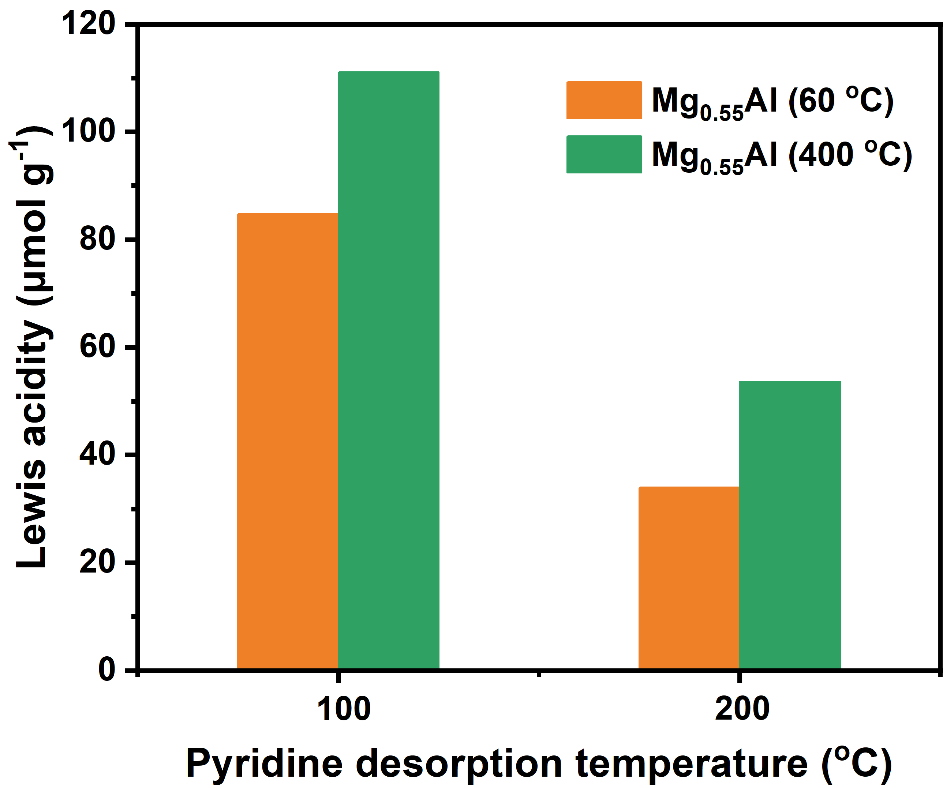
*

**Figure S7.** The concentrations of Lewis acid of Mg_0.55_Al (60 ^o^C) and Mg_0.55_Al (400 ^o^C).

**References**

[1] a)Q. Wang, D. O'Hare, “Large-scale synthesis of highly dispersed layered double hydroxide powders containing delaminated single layer nanosheets,” *Chemical Communications* (2013), *49*, 6301. 10.1039/c3cc42918k; b)X. Zhu, T. Ge, F. Yang, M. Lyu, C. Chen, D. O'Hare, R. Wang, “Efficient CO_2_ capture from ambient air with amine-functionalized Mg-Al mixed metal oxides,” *Journal of Materials Chemistry A* (2020), *8*, 16421. 10.1039/d0ta05079b; c)M. Zhao, J. Xiao, W. Gao, Q. Wang, “Defect-rich Mg-Al MMOs supported TEPA with enhanced charge transfer for highly efficient and stable direct air capture,” *Journal of Energy Chemistry* (2022), *68*, 401. 10.1016/j.jechem.2021.12.031

[2] L. Huang, J. Wang, Y. Gao, Y. Qiao, Q. Zheng, Z. Guo, Y. Zhao, D. O'Hare, Q. Wang, “Synthesis of LiAl_2_-layered double hydroxides for CO_2_ capture over a wide temperature range,” *Journal of Materials Chemistry A* (2014), *2*, 18454. 10.1039/c4ta04065a

[3] J. Fan, X. Yue, Y. Liu, D. Li, J. Feng, “An integration system derived from LDHs for CO_2_ direct capture and photocatalytic coupling reaction,” *Chem Catalysis* (2022), *2*, 531. 10.1016/j.checat.2022.01.001

[4] M. Lyu, J. Zheng, C. Coulthard, J. Ren, Y. Zhao, S. C. E. Tsang, C. Chen, D. O'Hare, “Core-shell silica@Cu_x_ZnAl LDH catalysts for efficient CO_2_ hydrogenation to methanol,” *Chemical Science* (2023), *14*, 9814. 10.1039/d3sc02205f

[5] Y. Gao, Z. Zhang, J. Wu, X. Yi, A. Zheng, A. Umar, D. O'Hare, Q. Wang, “Comprehensive investigation of CO_2_ adsorption on Mg–Al–CO_3_ LDH-derived mixed metal oxides,” *Journal of Materials Chemistry A* (2013), *1*. 10.1039/c3ta13039h

[6] J. Wang, Y. Zhang, J. Si, W. Zhang, Q. Liang, W. Li, B. Jin, S. Miao, “Structural engineering of NiFe-Layered double hydroxides and halloysite composites for efficient CO_2_ capture,” *Chemical Engineering Journal* (2023), *463*. 10.1016/j.cej.2023.142502

[7] W. Guo, C. Dun, C. Yu, X. Song, F. Yang, W. Kuang, Y. Xie, S. Li, Z. Wang, J. Yu, G. Fu, J. Guo, M. A. Marcus, J. J. Urban, Q. Zhang, J. Qiu, “Mismatching integration-enabled strains and defects engineering in LDH microstructure for high-rate and long-life charge storage,” *Nature Communications* (2022), *13*, 1409. 10.1038/s41467-022-28918-0

[8] S. Zheng, C. Song, M. C. Curria, Z. J. Ren, C. E. White, “Ca-Based Layered Double Hydroxides for Environmentally Sustainable Carbon Capture,” *Environmental Science & Technology* (2023), *57*, 17212. 10.1021/acs.est.3c03742

[9] B. Ge, C. Chen, Z. Gan, X. Zhu, Y. Miao, Y. Wang, T. Ge, D. O’Hare, R. Wang, “Scalable Synthesis of Amine-Grafted Ultrafine Layered Double Hydroxide Nanosheets with Improved Carbon Dioxide Capture Capacity from Air,” *ACS Sustainable Chemistry & Engineering* (2023), *11*, 9282. 10.1021/acssuschemeng.3c01183

[10] B. Ge, C. Chen, Y. Xu, S. Roberts, M. Zhang, Q. Shao, D. O’Hare, X. Zhu, “Enhancing adsorbent performance for direct air capture of CO_2_ by in-situ amine-grafting of layered double hydroxides,” *Chemical Engineering Journal* (2024), *500*. 10.1016/j.cej.2024.156782

[11] N. Tang, T. He, J. Liu, L. Li, H. Shi, W. Cen, Z. Ye, “New Insights into CO_2_ Adsorption on Layered Double Hydroxide (LDH)-Based Nanomaterials,” *Nanoscale Research Letters* (2018), *13*, 55. 10.1186/s11671-018-2471-z
